# Trojan Horse Strategy: How Biomimetic Nanomedicine Remodels the Tumor Microenvironment

**DOI:** 10.1002/advs.202519213

**Published:** 2026-01-12

**Authors:** Wanrong Wang, Siqi Mu, Juan Zhang, Guisong Shan, Xueqian Li, Ran Wang, Tao Guo, Xiaoyan He

**Affiliations:** ^1^ School of Life Sciences Anhui Medical University Hefei P. R. China; ^2^ Department of Respiratory and Critical Care Medicine The First Affiliated Hospital of Anhui Medical University Hefei P. R. China; ^3^ Department of Endocrinology Sir Run Run Shaw Hospital Zhejiang University School of Medicine Hangzhou Zhejiang P. R. China; ^4^ Department of Thyroid and Breast Surgery The First Affiliated Hospital of Anhui Medical University Anhui Public Health Clinical Center Hefei Anhui P. R. China

**Keywords:** biomimetic nanomedicines, cancer immunotherapy, cell membrane camouflage, tumor microenvironment

## Abstract

Biomimetics is an interdisciplinary field that involves studying the structures, functions, principles, and behaviors of biological systems to draw inspiration and apply this knowledge to technological innovation and engineering design, thereby addressing complex challenges. ​​Biomimetic nanomedicine​​ represents a specific application of biomimetics within the realm of nanomedicine, where biological components are mimicked to construct sophisticated nanodrug delivery systems. These biomimetic nanosystems exhibit multiple unique advantages, including ​​targeted delivery to cells within the tumor microenvironment (TME)​​, ​​prolonged in vivo circulation time​​, ​​enhanced antigen/adjuvant loading capacity​​, and ​​high biocompatibility​​. These properties collectively ​​enhance the efficacy of chemotherapy, radiotherapy, immunotherapy, and photodynamic therapy (PDT)​​ by improving tumor‐specific targeting and reducing off‐target toxicity, thereby establishing biomimetic nanomedicines as ​​a promising platform for reprogramming the TME​​. This review highlights the ​​categorization​​, ​​design principles​​, and ​​manufacturing strategies​​ of biomimetic nanodrugs, providing a ​​systematic review​​ of the interplay between the TME and ​​biomimetic nanomedicines​​, and elucidating how their interaction potentiates tumor‐killing efficacy​​. Despite encouraging progress in biomimetic nanomedicines, challenges remain in their clinical translation, including biosafety concerns, scalable manufacturing processes, and optimal drug delivery efficiency. Advances in nanotechnology and precision engineering offer promising avenues for developing personalized biomimetic nanomedicines.

## Graphical abstract

This review focuses on biomimetic nanomedicines for tumor microenvironment (TME) remodeling, covering their diverse biomimetic types, design principles, and mechanisms of immune cell reprogramming and reversal of immunosuppressive microenvironments, with particular emphasis on their application in synergistic immunotherapy. Given the current key clinical translation barriers for nanomedicines, new insights are proposed for future research directions.

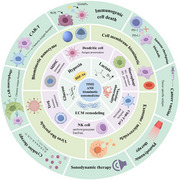

## 1 Introduction

**FIGURE 1:**
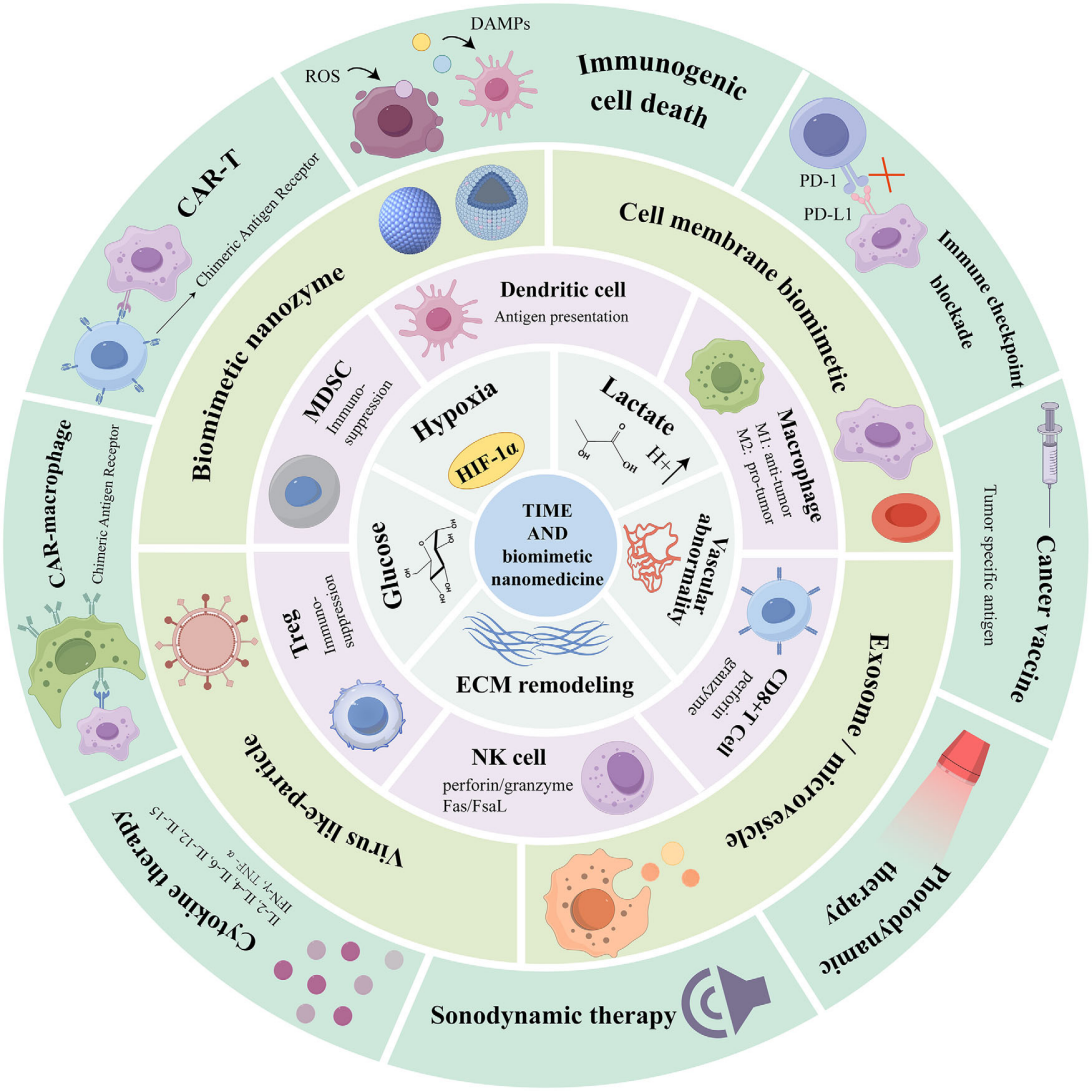
Biomimetic nanomedicines in the tumor microenvironment and applied to tumor immunotherapy.

This review systematically outlines the functional characteristics of key immune cells within the tumor microenvironment (TME), with a focus on the design principles and fabrication technologies of biomimetic nanomedicines [1]. It also highlights recent advances in their application for regulating the TME, while discussing their therapeutic potential in cancer and the challenges associated with clinical translation [2]. These insights provide a ​​theoretical foundation and novel perspectives​​ for developing next‐generation antitumor combination therapies (Figure 1).

Over the past decades, antitumor therapies have achieved ​​transformative breakthroughs​​, rapidly advancing from basic research to clinical applications [3]. Photodynamic therapy (PDT)​​ triggers photochemical reactions within tumor tissues, generating reactive oxygen species (ROS) that directly kill tumor cells or disrupt the tumor vascular system. ​​Immune checkpoint blockade (ICB) therapy​​ is currently applicable to dozens of solid tumors, while ​​chimeric antigen receptor (CAR)‐T cell therapy​​ has demonstrated remarkable efficacy against hematologic malignancies, significantly improving overall survival in advanced patients [4, 5]. Despite these successes, the therapeutic potential of these approaches remains substantially limited by challenges such as ​​suboptimal targeting​​, ​​drug resistance​​, and ​​significant adverse effects​​.

The TME represents a dynamic interface where tumor cells engage with the host immune system. This complex milieu comprises diverse immune cell populations, cytokines, vasculature, and extracellular matrix (ECM) components, collectively governing tumor progression, metastasis, and therapeutic resistance [6]. Interactions between distinct immune cell subsets and tumor cells critically dictate the functional state of the TME. The process of tumor angiogenesis consumes substantial oxygen and metabolic substrates, and the aberrant vasculature creates a physical barrier that restricts the infiltration of effector T cells into the TME [7]. Factors such as lactic acid accumulation, hypoxia, and immunosuppressive cell populations compromise effector activity, ultimately facilitating tumor escape [8, 9]. The pervasive immunosuppressive mechanisms and physical barriers inherent to the TME significantly limit the clinical efficacy of current therapies. Consequently, strategies for the ​​precise modulation of the TME​​ to reverse the ​​tumor‐killing suppressed state​​ have become pivotal in advancing ​​cancer immunotherapy.

**FIGURE 2:**
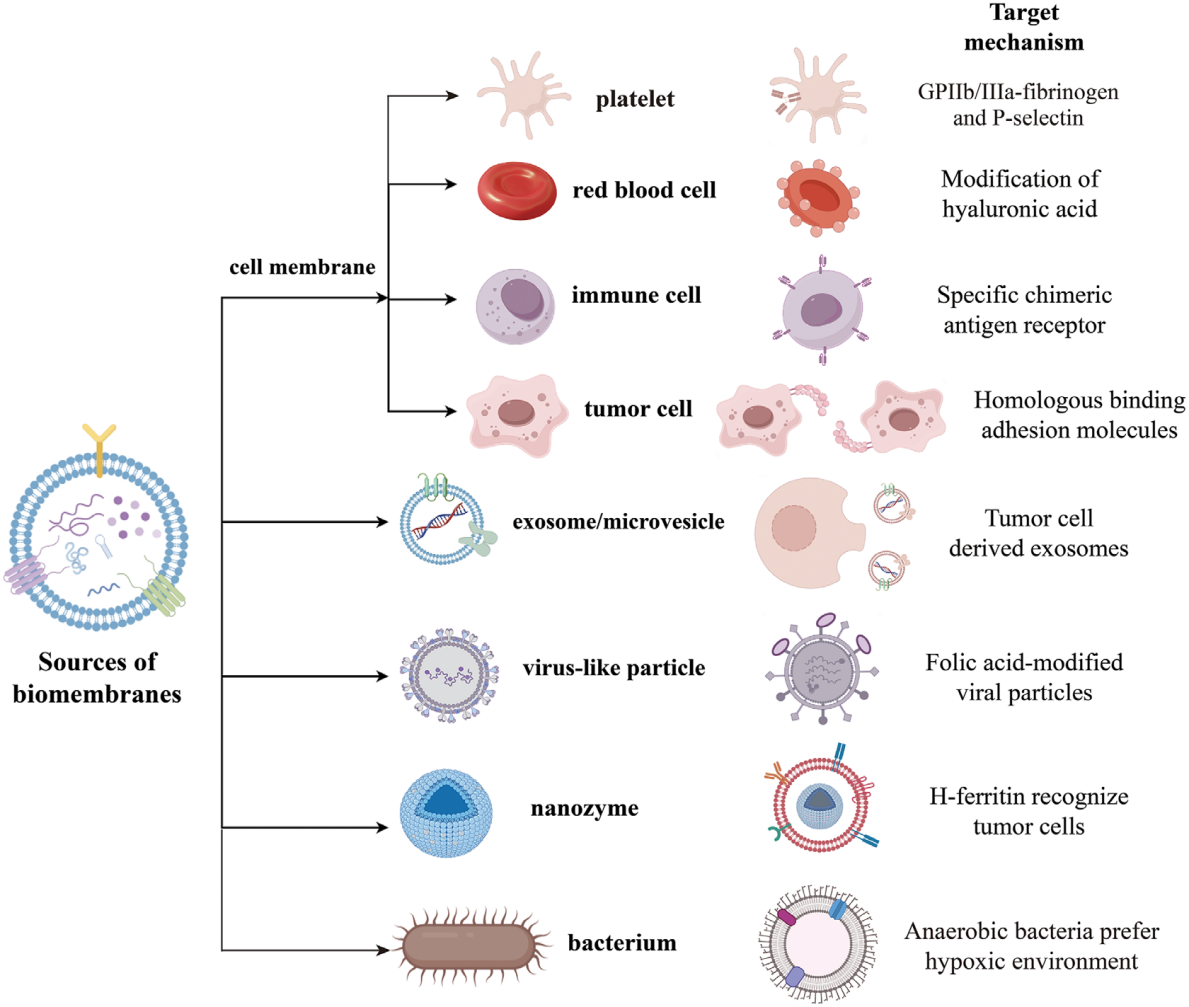
Representative sources and mechanisms of biomimetic nanomedicines targeting tumors.

Nanomedicine draws inspiration from natural biology, giving rise to biomimetic nanomedicine. By mimicking the structure and function of natural biological entities, biomimetic nanomedicines evade host immune surveillance through “self” camouflage. They actively return to tumor tissues via conserved recognition mechanisms to precisely deliver their payloads [10]. These systems can be broadly categorized into: cell membrane‐derived platforms (e.g., erythrocyte, tumor cell, or immune cell membrane coatings) [3, 11], extracellular vesicle‐inspired designs (e.g., exosomes, microvesicles) [12, 13], and pathogen‐mimetic constructs (e.g., viruses, bacteria) [14, 15]. By mimicking endogenous components, biomimetic nanomedicines enhance tumor targeting, evade immune clearance, and respond to microenvironmental cues. This enables efficient therapeutic delivery, amelioration of tumor hypoxia and acidosis, and remodeling of aberrant vasculature and stroma, thereby potentiating conventional therapies like radiotherapy and chemotherapy (Figure 2) [10, 16]. Crucially, these nanomedicines can directly reprogram immune cell function within the TME, restoring antitumor immunity. Specific mechanisms include modulating tumor‐associated macrophage (TAM) polarization, activating effector T cells, depleting immunosuppressive cells, and blocking PD‐L1‐mediated evasion, collectively enhancing tumor immune clearance [17, 18]. These nanomedicines act like ancient Trojan horses, concealing therapeutic payloads within biomimetic shells to deceive the host's immune surveillance system. They infiltrate deep into the tumor's fortress before launching a lethal assault on cancer cells.

Despite numerous reviews on biomimetic nanomedicines or TME regulation, most studies either focus on technical details of nanomedicine preparation or emphasize biological characteristics of the TME, lacking systematic integration of biomimetic design with tumor microenvironment regulation mechanisms. This review dissects the functional roles of key immune cell subsets within the TME and their interaction mechanisms. Centering on the Trojan horse strategy as a core thread, it connects different types of biomimetic nanomedicines, elucidating their design logic of disguise‐penetration‐destruction, and thoroughly analyzes their potential in cancer immunotherapy. Furthermore, this paper outlines the design principles for biomimetic nanomedicines and addresses translational challenges such as biosafety, scalable production, and delivery efficiency. These insights lay the foundation for developing next‐generation combination immunotherapies targeting the TME.

## 2 Tumor Microenvironment

This section first categorizes key immune cell populations within the TME and their functional roles, followed by an elaboration of core immune regulatory mechanisms. Understanding these components and their characteristics establishes a foundational framework for subsequent TME reprogramming research based on biomimetic nanomedicine.

### 2.1 Immune Cell Populations

#### 2.1.1 Antigen‐Presenting Cells

Tumor cells typically lack major histocompatibility complex (MHC) class II molecules and co‐stimulatory molecules. Consequently, cancer neoantigens displayed on MHC class I are recognized, processed, and cross‐presented to CD8+ T cells by professional APCs (macrophages, dendritic cells (DCs), and B cells), activating tumor cell killing [19]. Among APCs, DCs exhibit superior antigen‐presenting capacity and are essential for initiating and sustaining antitumor immunity [20, 21].

Chemokines from tumor and immune cells recruit DCs into the TME. DCs phagocytose tumor antigens, process them, and load them onto MHC‐I for presentation to T cells within the TME or in draining lymph nodes, activating cytotoxic CD8+ T cells [22]. DCs also secrete cytokines like interleukin (IL)‐12 to enhance T cell and natural killer (NK) cell antitumor activity [23]. Recent advances show CCR7+ CD141+ DCs in human tumors transport antigens to lymph nodes, directly stimulating CD8+ T cell activation. CCR7 deletion impairs T cell priming and accelerates tumor growth [24]. TME DCs also highly express PD‐L1, which binds PD‐1 on T cells to inhibit immune responses [25]. PD‐L1‐deficient DCs exhibit increased chemosensitivity and ferroptosis susceptibility, reducing DC numbers and diminishing tumor‐specific T cell activation and antitumor responses. PD‐L1 upregulates the antioxidant SLC7A11, inhibiting lipid peroxidation and protecting DCs from chemotherapy toxicity [26].

TME factors can limit DC growth, recruitment, development, and antigen presentation. Tumor‐derived TLR2 ligands stimulate DC autocrine IL‐6/IL‐10, promote pre‐DC differentiation into immunosuppressive DCs, and inhibit IL‐12 production, preventing T cell activation. Conversely, TLR2 blockade enhances DC antitumor effects and immunotherapy efficacy [27]. In HCC, β‐catenin activation reduces tumor CCL5 expression, impairing CD103+ DC recruitment and antigen‐specific CD8+ T cell activation, thereby promoting immune evasion [28]. Preserving DC antigen processing/presentation within the TME is crucial for activating antigen‐specific T cell immunity. Understanding DC subset diversity and TME‐mediated constraints is vital for developing precision immunotherapies.

Macrophages, derived from blood monocytes migrating into tissues, participate in all tumor stages [29]. Early in cancer, cytokines/chemokines recruit macrophages into the tumor stroma. These cells directly phagocytose tumor cells and present tumor antigen peptides via MHC to T cells in the TME, promoting T cell activation and antitumor immunity with co‐stimulatory molecules [30]. However, TME macrophages can also enhance tumor proliferation, angiogenesis, metastasis, and inhibit CD8+T cell recruitment/activation [31, 32]. This functional plasticity allows distinct polarization: tumor necrosis factor (TNF)‐α or interferon (IFN)‐γ stimulation induces classically activated M1 macrophages, secreting TNF‐α, IL‐6, and ROS to promote inflammation and tumor killing [33]. IL‐4, IL‐10, and IL‐13 induce alternatively activated M2 macrophages with weaker antigen presentation; they secrete inhibitory cytokines (IL‐10, TGF‐β) and vascular endothelial growth factor (VEGF) to suppress immunity, stimulate angiogenesis, and accelerate tumor growth [34, 35]. Thus, macrophages represent a double‐edged sword. Breaking the TME immunosuppressive cycle to enhance their antitumor effects is key for macrophage‐targeted therapies.

In summary, APCs critically regulate anti‐cancer immune responses within the complex TME system. Immunosuppressive factors often impair APC function, enabling immune escape and tumor progression, posing challenges for APC‐based immunotherapy [36]. Strategies to enhance APC function are actively pursued to overcome these obstacles.

#### 2.1.2 Effector Cells

Effector cells directly kill tumor cells in the TME, primarily CD8+ T lymphocytes, NK cells, and M1 macrophages [37]. CD8+T lymphocytes (Cytotoxic T Lymphocytes, CTLs) are central to antitumor immunity and adoptive cell therapy. Their tumor infiltration correlates with favorable prognosis [38]. APC‐activated CD8+ T cells enter the TME via chemokines. Their TCR binds tumor cell surface MHC‐I/antigen peptide complexes, initiating killing [39]. CTLs directly kill via perforin/granzyme‐containing cytotoxic granules or indirectly via cytokines (IFN‐γ, TNF‐α) [40]. CTL‐expressed Fas ligand (Fas‐L) also engages Fas on cancer cells, inducing caspase‐dependent apoptosis [41]. Despite intratumoral CTL infiltration, progression often occurs due to TME‐induced CTL dysfunction [42]. Tumors evade immunity by downregulating MHC and upregulating immune checkpoint ligands (PD‐L1, PD‐L2, TIM‐3) and inhibitory cytokines (TGF‐β, IL‐10), impairing CTL function [43]. While CTL‐based therapies show efficacy in some cancers, preventing T cell exhaustion and sustaining cytotoxicity are key therapeutic goals.

NK cells, crucial innate immune components, limit cancer growth similarly to CTLs [44]. Unlike CTLs, NK cells lack antigen‐specific receptors, or APC dependence. They non‐specifically kill tumors via surface inhibitory/activating receptors [45]. NK cells primarily employ a “missing‐self” mechanism: normal cell MHC‐I binding to NK inhibitory receptors blocks activation, but tumor cells downregulate MHC‐I (evading CTLs). This loss of inhibition combined with dominant activating signals triggers NK cell release of perforin/granzymes, inducing tumor lysis [46]. Antibody‐dependent cellular cytotoxicity (ADCC) is another key mechanism. Antibody binding (Fab to tumor antigen, Fc to CD16 on NK cells) activates cytotoxic granule release [47]. Although NK cell immunotherapy shows promise, TME factors in leukemia (hypoxia, low glucose, adenosine, lactate) impair NK cell metabolism and activity, promoting tumor progression [48, 49]. Overcoming these limitations is essential for maximizing NK cell potential.

Given TME constraints on effector cell function, strategies to restore activity include immune checkpoint inhibitors, cytokines/tumor vaccines to enhance proliferation/recognition, and engineering effector cells for improved targeting/killing [50]. These approaches aim to overcome TME suppression and enhance immunotherapy efficacy.

#### 2.1.3 Immunosuppressive Cell

TME immunosuppressive cells, including TAMs, regulatory T cells (Tregs), and myeloid‐derived suppressor cells (MDSCs), could weaken antitumor immunity via inhibitory cytokines, immune checkpoint expression, metabolic competition, and direct effector cell inhibition [51].

The 2025 Nobel Prize in Physiology or Medicine was awarded to Mary E. Brunkow, Fred Ramsdell, and Shimon Sakaguchi for their discoveries in peripheral immune tolerance. These winners have identified the security of the immune system, Tregs, which can prevent immune cells from attacking our own bodies. But they can also become a rebel force to prevent antitumor immunity. Tregs comprise natural Tregs (nTregs, thymus‐derived) and induced Tregs (iTregs, peripherally differentiated) [52]. TME Tregs are primarily iTregs, defined by Foxp3 expression and potent immunosuppression, limiting inflammation, and antitumor immunity [53]. Increased tumor Treg infiltration correlates with poor prognosis [7]. Chemokine receptors guide Treg recruitment to the TME via tumor‐derived ligands (CXCL12, CCL17, CCL22) [54]. Hypoxia‐induced hypoxia‐inducible factor (HIF)‐1 promotes CD4+ T cell differentiation into immunosuppressive Tregs via Foxp3 induction. Foxp3 drives constitutive CTLA‐4 expression. CTLA‐4 binds CD80/CD86, blocking co‐stimulation, and removes these molecules from APCs via trans‐endocytosis, inhibiting CD28 signaling [55]. Tregs also secrete inhibitory cytokines (TGF‐β, IL‐10, IL‐35) and directly kill CD8+ T cells and NK cells via granzymes/perforin, facilitating immune escape [56]. Treg‐targeting strategies (blocking migration, depletion, cytokine inhibition) aim to improve patient outcomes.

Immunosuppressive TAMs, predominantly M2‐polarized, promote tumor growth and correlate with poor prognosis [57]. TAM‐produced IL‐10 inhibits antitumor cytokines (IL‐12, IFN‐γ), impairing naïve T cell differentiation and cytotoxic CD8+ T/NK cell function [58]. Tumor cell‐derived TNF‐α induces pro‐angiogenic factors (e.g., VEGF) in TAMs [59]. In CRC, CD163+ TAMs secrete IL‐6, inducing epithelial‐mesenchymal transition via the STAT3/miR‐506‐3p/FoxQ1 pathway, enhancing metastasis. Elevated tumor CCL2 further recruits TAMs, creating a vicious cycle [60]. Targeting TAMs (inhibiting recruitment, promoting M1 polarization or M2‐to‐M1 conversion) is a promising therapeutic approach [61].

Hematopoietic stem cells normally differentiate into immature myeloid cells (IMCs), precursors to DCs, macrophages, and granulocytes [62]. Pathological conditions, especially the TME, impede IMC maturation, resulting in MDSCs with potent immunosuppressive functions [63]. Hypoxic TME upregulates MDSC PD‐L1 via HIF‐1α; PD‐L1/PD‐1 binding induces T cell dysfunction. Hypoxia also increases MDSC secretion of IL‐10 and TGF‐β1. MDSCs deplete L‐arginine, essential for T cell function [64]. Upregulated iNOS metabolizes L‐arginine to L‐citrulline and NO; NO disrupts IL‐2R signaling and nitrates TCRs, impairing antigen recognition [65]. MDSC‐derived ROS and peroxynitrite (ONOO^−^) further damage T cell viability [66]. MDSC‐targeting therapies are under development, necessitating deeper understanding of their biology.

Characterizing immunosuppressive cells enables strategies to block their function, reverse TME suppression, and restore effector cell antitumor activity [67]. The following section details the characteristics of the TME and their cross‐talk with immune cells.

### 2.2 Mechanisms of TME Immunoregulation

#### 2.2.1 Metabolic Regulation

The Warburg effect is an important metabolic feature of tumor cells, which refers to the fact that tumor cells, even in the presence of sufficient oxygen, preferentially choose to obtain energy through glycolysis, a process that produces large amounts of lactate [68]. This metabolic shift creates an acidic milieu that simultaneously promotes tumor progression and profoundly suppresses antitumor immunity. Specifically, it impairs the proliferation, activation, and cytokine secretion of CD4+ and CD8+ T cells. Meanwhile, it induces mitochondrial dysfunction and apoptosis in liver‐resident NK cells via ROS overproduction [69, 70]. In breast cancer models, lactate drives TAMs polarization toward pro‐tumorigenic M2 phenotypes via ERK/STAT3 signaling while enhancing cancer cell proliferation and angiogenesis [71], which further expands immunosuppressive populations by promoting MDSC development and Tregs differentiation while inhibiting dendritic cell maturation and NK cell cytotoxicity [72]. Furthermore, targeting the accelerated proliferation and metabolic rates of tumor cells, interfering with their energy metabolism has emerged as a novel approach to disrupt homeostasis and induce ferroptosis in tumor cells, thereby opening new avenues for clinical cancer treatment [73].

Complementing lactate's effects, dysregulated tryptophan metabolism establishes additional immunosuppressive circuits. Tumors overexpress indoleamine‐2,3‐dioxygenase (IDO) and tryptophan‐2,3‐dioxygenase (TDO), catabolizing this essential amino acid into immunosuppressive metabolites. IDO recruits myeloid‐derived suppressor cells in Treg‐dependent fashion, while TDO‐mediated tryptophan depletion starves cytotoxic lymphocytes and forces dendritic cells into tolerogenic states [74, 75]. The resulting kynurenine metabolites activate the aryl hydrocarbon receptor (AhR), creating a feed‐forward loop that amplifies IDO/TDO expression while inducing inflammatory cytokines like IFN‐γ and IL‐6 [76]. These metabolic abnormalities directly impair the function of effector T cells and NK cells, creating an inhibitory tumor microenvironment that impedes the efficacy of immunotherapy.

#### 2.2.2 Hypoxia

Normal cells have difficulty surviving in hypoxic environments, but most TMEs are characterized by a hypoxic state and are significantly affected immune cells in the TME [77]. Crucially, HIF‐1α activation under low oxygen significantly increases PD‐L1 expression on MDSCs, macrophages, and DCs. This upregulation directly inhibits T cell activation and promotes their apoptosis. Furthermore, hypoxia drives the recruitment of immunosuppressive Tregs, particularly in HCC, by inducing the chemokine CCL28; knocking down HIF1A reduces CCL28 and consequently Treg infiltration [78]. In addition, as a transcriptional regulator, HIF promotes the expression of various angiogenic factors such as VEGF, stromal cell‐derived factor 1, and angiopoietin 2 in tumors [79]. HIF also inhibits the oxidative phosphorylation process in the mitochondria by activating the genes encoding pyruvate dehydrogenase (PDH) kinase and lactate dehydrogenase, allowing glycolysis to become the main mode of energy generation in cancer cells [80]. Recently, cancer therapies targeting HIF have received increasing attention to inhibit tumor development by inhibiting HIF activity through various pathways. For example, the HIF1A inhibitor PX‐478 has been shown to inhibit tumor growth, promote apoptosis in cancer cells, and reduce PD‐L1 expression [81]. Further understanding of the regulatory effects of hypoxia and HIF on cancer cells and exploring specific regulatory mechanisms will be very helpful in developing therapeutic targets.

#### 2.2.3 Extracellular Matrix Remodeling

The ECM of TME is primarily composed of collagen, hyaluronic acid, fibronectin, and laminin. Dense networks of collagen fibers create formidable barriers that physically obstruct immune cell infiltration, particularly into tumor cores, while hyaluronic acid binds to tumor cell surface receptors to promote proliferation and inhibit apoptosis [82]. In ovarian cancer, elevated collagen deposition actively prevents T cells from migrating from the stromal periphery to engage cancer cells directly, severely limiting their antitumor function [6, 83]. Simultaneously, the ECM undergoes constant remodeling influenced by cellular activity within the TME. Notably, tumor cells can co‐opt immune cells like macrophages. HER2‐positive breast cancer cells stimulate macrophages to overexpress matrix metalloproteinase (MMP) 11. This enzyme degrades ECM components, facilitating not only cancer cell invasion but also indirectly promoting angiogenesis [84]. The resulting ECM breakdown and reorganization further disrupt normal immune cell trafficking and positioning. In addition, the ECM actively influences tumor cells within the TME, further shaping cancer progression. Beyond its structural role, specific ECM components like hyaluronic acid can engage directly with tumor biology by binding TLR4 on colorectal cancer cells to drive proliferation while blocking apoptosis [85]. Furthermore, the basement membrane, a critical barrier against infiltration and spread, is systematically breached by tumors. Cancer cells secrete enzymes that degrade this membrane and other ECM constituents, actively enhancing their invasive potential [86]. Consequently, targeting specific ECM components or the enzymes that remodel them presents a promising strategy to disrupt this physical immunosuppression.

#### 2.2.4 Vascular Abnormality

**FIGURE 3:**
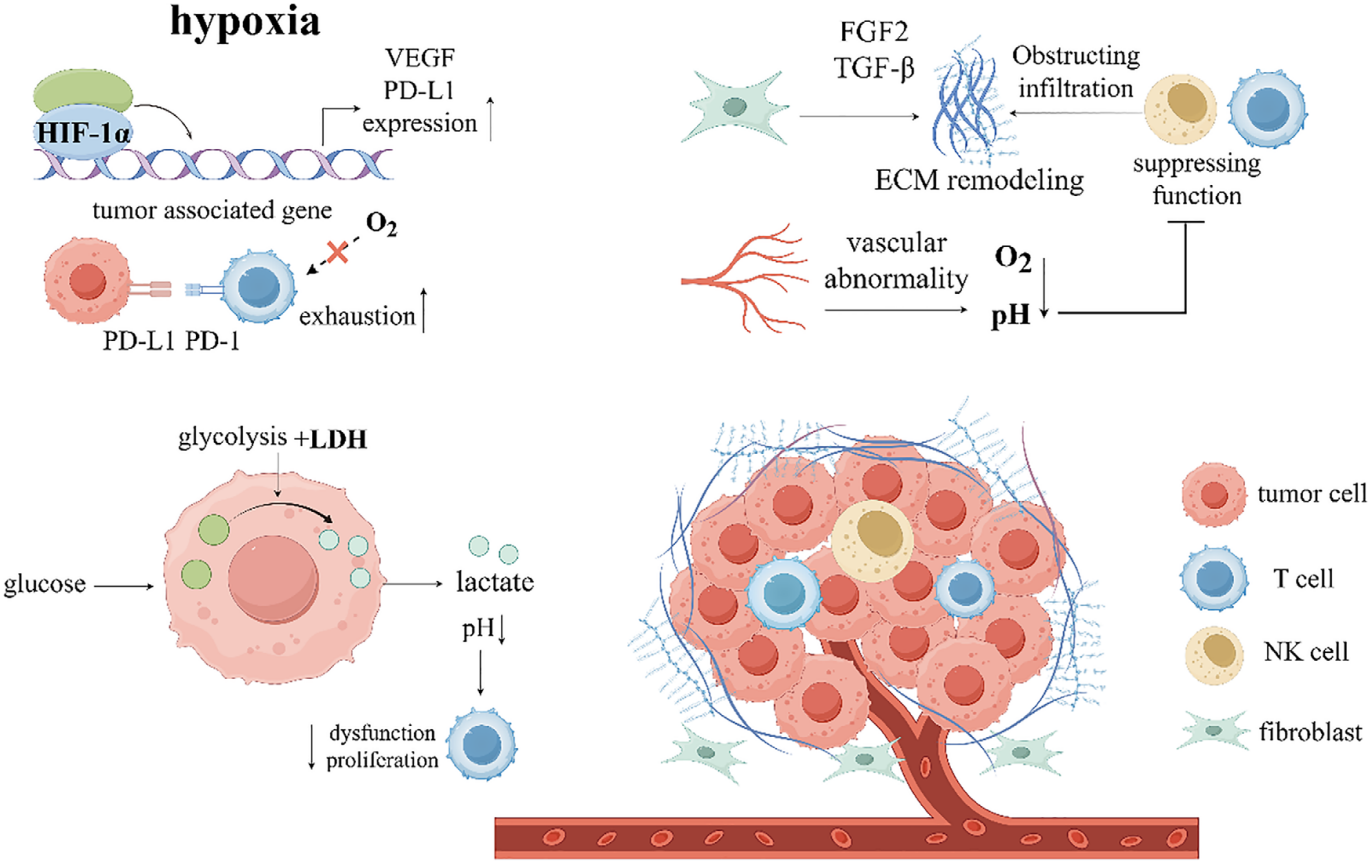
Lactate metabolism, hypoxia, ECM remodeling, vascular abnormalities, and immunosuppressive cells combine to form an immunosuppressive microenvironment.

Tumor vasculature is characterized by disorganized structure, increased permeability, and poor perfusion. This abnormality not only causes hypoxia and acidosis but also reduces the delivery of chemotherapeutic drugs and immune cells to the tumor core, forming a vascular barrier for antitumor therapy [87]. Critically, immune cells within the TME are active participants in this pathological vascularization. TAMs secrete potent pro‐angiogenic factors, including VEGF itself, fibroblast growth factor (FGF) 2, and epidermal growth factor (EGF), driving endothelial proliferation and new vessel formation [88]. Similarly, MDSCs contribute by releasing MMPs. MMP activity degrades the extracellular matrix, physically creating space for sprouting vessels and enhancing tumor cell invasion. Moreover, MMP‐9 specifically cleaves factors to increase the bioavailability of VEGF, further amplifying angiogenic signaling [89]. This immune‐facilitated angiogenesis establishes a self‐reinforcing cycle: therapies targeting VEGF/VEGFR often induce even greater hypoxia, paradoxically stimulating stronger pro‐angiogenic responses [90]. Understanding this intricate immune‐driven vascular network is therefore essential for developing more effective strategies. In addition, the abnormal vascular structure and function the tumor less responsive to treatment, and chemotherapeutic drugs cannot be delivered to the tumor locally, resulting in greatly reduced efficacy [91]. Antitumor angiogenesis and induction of its normalization are commonly used and effective tumor treatment strategies, and targeted drugs such as large molecule monoclonal antibodies against VEGF/VEGFR signaling pathway have been widely used in the clinic [90]. An in‐depth understanding of the regulatory mechanisms of angiogenic signaling pathways can provide new ideas for the development of more efficient therapies. Figure 3 summarizes the multidimensional immunosuppressive network of TME.

## 3 Biomimetic Nanomedicine

This section systematically elaborates on nanomedicine platforms constructed based on distinct bio‐inspired principles, encompassing five major categories: cell membrane‐camouflaged nanocarriers, extracellular vesicles (EVs), virus‐like particles (VLPs), nanozymes, and bacteria‐inspired nanosystems. The core of their biomimetic design lies in achieving intelligent responsiveness and efficient intervention within complex biological environments through the simulation and reconstruction of natural biological structures, components, or functions.

Specifically, cell membrane‐camouflaged nanoparticles (NPs) draw inspiration from biomembrane‐mediated intercellular recognition and communication mechanisms. By coating synthetic NP cores with natural cell membranes, such as those derived from erythrocytes, immune cells, or cancer cells, these systems not only preserve the complete surface proteome and bio‐interface characteristics of the source cells but are also endowed with core functionalities including immune evasion, tissue‐specific targeting, and bio‐adhesion. The biomimetic value of EVs resides in their full retention of the biocompatibility and inherent homing capabilities intrinsic to natural delivery systems. VLPs exemplify the biomimetic utilization of viral capsid proteins, which exhibit efficient spontaneous self‐assembly properties and sophisticated cellular invasion mechanisms. By retaining the structural proteins of viruses while eliminating their genetic material, these particles mimic the cellular attachment and internalization efficiency of native viruses, yet completely avoid risks associated with replication and pathogenicity, thereby offering an ideal platform for vaccine development and gene delivery. The biomimetic strategy underlying nanozymes involves simulating the catalytic centers and reaction mechanisms of natural enzymes. These nanostructures can be further functionalized with cell membrane coatings to enhance tumor cell targeting. Bacteria‐inspired NPs, meanwhile, leverage the innate tumor tropism and immune‐activating properties of microorganisms. Utilizing attenuated bacteria or their membrane components as carriers allows effective targeting of hypoxic tumor regions. Furthermore, pathogen‐associated molecular patterns present on these biomimetic systems can activate innate immunity, thereby reprogramming the immunosuppressive microenvironment.

These biomimetic strategies share the common feature of translating biological principles into feasible engineering solutions. Guided by the design philosophy of “learning from nature for therapeutic applications,” intelligent biomimetic nanosystems can actively adapt to biological environments, overcome physiological barriers, and execute complex tasks. Collectively, these platforms offer highly promising technical tools for reprogramming the TME.

### 3.1 Cell Membrane‐Camouflaged Nanomedicines

**FIGURE 4:**
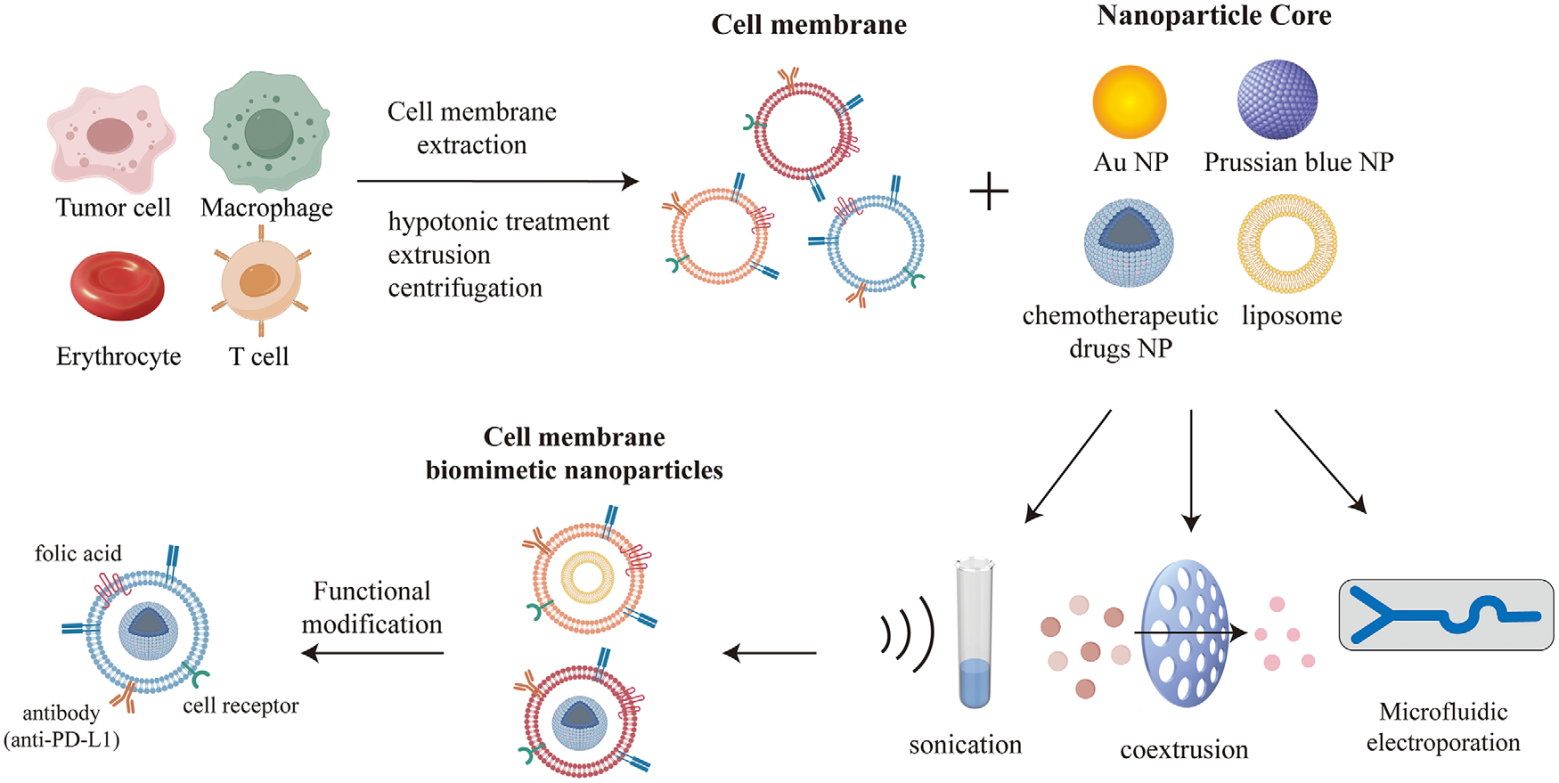
Process for the preparation of biomimetic nanomedicines.

Cell membrane‐camouflaged nanomedicines represent a bioinspired paradigm in which synthetic NPs are enveloped by naturally derived membranes, typically sourced from erythrocytes, neoplastic cells, or effector cells. This approach leverages conserved membrane architectures to confer biological functionality upon synthetic cores [92]. The fabrication process comprises three sequential stages: membrane isolation through hypotonic lysis combined with sucrose density gradient centrifugation for purification; vesicle formation via extrusion or sonication to generate nanoscale membrane vesicles; and core‐vesicle fusion using microfluidic extrusion or electroporation to achieve nanoparticle encapsulation [93]. Critically, the resulting constructs retain source cell‐specific surface proteomes, including CD47, integrins, and TCR complexes, which collectively mediate immune evasion, tissue tropism, and environmental responsiveness. These systems address inherent limitations of conventional nanocarriers, notably opsonization and rapid clearance by mononuclear phagocyte systems, exhibiting substantially prolonged plasma circulation compared to uncoated counterparts (Figure 4) [11].

Functional properties diverge according to membrane source: Erythrocyte‐based systems exploit CD47‐ signal‐regulating protein α (SIRPα) inhibitory signaling to evade phagocytosis while demonstrating extended circulation capabilities. Their limited intrinsic tumor targeting often requires antibody‐mediated functionalization for precise delivery [94]. Cancer cell membrane‐coated NPs inherently target homologous malignancies through conserved adhesion molecules such as Epithelial cell adhesion molecule and galectin‐3, showing enhanced tumor accumulation relative to conventional nanocarriers [95]. Tumor‐associated antigens (TAAs) on these membranes further facilitate DCs maturation and antigen‐specific T cell activation, creating a self‐adjuvant effect that amplifies immunogenic cell death (ICD). Immune cell membranes confer specialized functions: macrophage membranes enable inflammatory site homing through chemokine receptors and blood‐brain barrier penetration via specific receptor interactions, while T cell membranes utilize TCR‐mediated recognition for tumor‐specific delivery [96]. Hybrid systems combining membrane types demonstrate synergistic efficacy in metastasis targeting through combined homologous targeting and immune cell trafficking mechanisms [97].

Despite promising preclinical outcomes, clinical translation encounters three primary barriers: membrane protein instability during processing leading to functional compromise; manufacturing scalability limitations due to batch heterogeneity and suboptimal fusion efficiency; and persistent immunogenicity concerns from residual cellular components [98]. Addressing these requires integrated strategies: implementation of membrane‐stabilizing agents and gentle processing techniques; development of continuous manufacturing platforms compliant with quality standards; and utilization of autologous sources or epitope‐editing technologies to mitigate immune recognition. Future advancement should prioritize stimuli‐responsive membrane systems incorporating environmental triggers and combinatorial approaches with immune modulators. Overcoming these challenges remains pivotal for transforming biomimetic nanoplatforms from experimental prototypes to clinically viable therapeutics. Table 1 summarizing the properties of different sources of biomimetic nanomedicines and their applications in tumor therapy.

**TABLE 1:** Characterization and application of multiple biomimetic nanomedicines.

Biomimetic membrane	Diameter	Drug‐loaded	Cancer type	Targeting tumors mechanism	Effects	Limitations	Refs.
RBC membrane	120 nm	Photosensitizing AIEgen and immunologic adjuvant Poly (I : C)	Melanoma	Enhanced permeability and retention effect	Increases levels of immune factors (IL‐1α, IL‐6, TNF‐α, and IFN‐γ). Promotes the release of tumor antigen, activating T cells	Lack of natural active tumor targeting	[265]
	140 nm	Gamabufotalin (CS‐6)	Breast cancer	Modification of hyaluronic acid enables targeted accumulation at tumor sites	Prolong the blood circulation TME of the drug, enhance the ability to evade immune surveillance, and improve the efficacy of chemo/photothermal therapy	Lack of precise drug release capability	[266]
Cancer cell membrane	326.4 nm	Imiquimod, docetaxel, Prussian blue	Breast cancer	Homologous binding adhesion molecules (EpCAM and galectin‐3) can recognize and bind to cancer cells	Promotes DCs maturation and produces a variety of pro‐inflammatory cytokines. Enhance the efficacy of photothermal therapy	Higher risk of biosafety and immunogenicity	[267]
	247 nm	Doxorubicin, gold NPs	Breast cancer	4T1 cell membrane coating increases affinity for tumor cells, preferentially entered homologous tumor cells	Induce immunogenic cell death in breast cancer cells, promote DC maturation, and activate immune response	Requires homologous cancer cell membranes, complex and costly to prepare	[268]
Macrophage membrane	212 nm	Acoustic sensitizer IR780 and catalase	Breast cancer	Cancer cells mediate the uptake of NPs through actin, as well as caveolin and clathrin‐dependent endocytic pathways	Enhance the efficacy of sonodynamic therapy (SDT), alleviating tumor hypoxia and downregulated HIF‐1α	Macrophage membrane separation process tends to result in loss of protein activity	[269]
	168 nm	Sonosensitizer Ce6 and JQ1 (a bromo‐domain protein 4 (BRD4) inhibitor which can down‐regulate PD‐L1)	Glioblastoma	Recognition of α4 and β1 integrins and the vascular cell adhesion molecule‐1	Induces apoptosis, activates the immune system, and PD‐L1 checkpoint inhibitor JQ1 inhibits tumor growth	Difficulty of macrophage expansion in vitro and low efficiency of membrane extraction	[270]
T cell membrane	165.9±1.0 nm	Paclitaxel	Gastric cancer	The interaction between LFA‐1 and ICAM‐1 contributes to the localization of T cell in tumors	Chemotherapy drugs are combined with local low‐dose irradiation therapy to inhibit tumor growth	Receptor expression in the membrane is affected by the activation state, leading to unstable targeting efficiency	[132]
	107 nm	AIE NPs	Glioblastoma	Specific chimeric antigen receptor CD133/EGFR is expressed on cell membranes to target cancer cells	Cross the blood‐brain barrier, generating photothermal effect to induce potent apoptosis in tumor cells	T cell membrane surface proteins are susceptible to denaturation or shedding during the preparation process	[271]
DC membrane	127.9±1.5 nm	Rapamycin	Glioma	Homologous tumor cells can be targeted through the tumor antigens from tumor cell lysate endocytosis by DCs	Promotes DCs maturation to activate immune cells and induce the subsequent immune responses	DCs need to be induced to mature in vitro, and the process of extracting membranes is complex and TME‐consuming	[272]
Exosomes	150 nm	Doxorubicin	Liver cancer, breast cancer, melanoma	Tumor cell‐derived exosomes facilitate tumor targeting	Enhance the penetration ability and the accumulation in tumors, and enhance the cytotoxicity of drugs	Complex purification process, low yield, and high cost	[273]
Microvesicle	792 nm	Bcl2 siRNA and paclitaxel	Breast Cancer	Folate is modified onto the membrane of MVs	Synergistic treatment of tumors with Bcl2 siRNA and paclitaxel carried in microvesicles	Low drug loading efficiency and release controllability	[274]
Virus‐Like Particles	27 nm	ODN1826 (CpG‐ Oligodeoxynucleotides)	Colon cancer, melanoma		Enhance the in vivo antitumor activity of macrophages in tumors	Inadequate target specificity and controlled drug release	[275]
	84.1 ± 16.5 nm	Paclitaxel	Breast Cancer	Folic acid‐modified viral particles enhance targeting of Folic acid receptor‐positive tumors	Enhanced tumor targeting ability and water solubility of Paclitaxel and reduced toxicity to normal tissues using adenoviral vectors	Higher risk of immunogenicity and toxicity	[276]
Nanozyme	100 ± 10 nm		Colon cancer, liver cancer	H‐ferritin NPs specifically recognize tumor cells expressing high levels of the HFn receptor	Nanozyme using nitrogen‐doped porous carbon nanospheres can boost reactive oxygen species generation in a tumor‐specific manner	Inorganic nanomaterials may accumulate in the liver with long‐term toxicity risks	[277]
	44.67 ± 1.29 nm	photosensitizer chlorin e6 (Ce6)	Breast Cancer		Catalyzes the decomposition of hydrogen peroxide in tumors, provides oxygen for photodynamic action, and alleviates hypoxia	High production costs and a lack of targeting	[278]

#### 3.1.1 Erythrocyte Membranes‐Camouflaged Nanomedicines

Red blood cells (RBCs), the most abundant cells in the human circulation, have a long history of being developed for drug delivery and are ideal carriers of a wide range of biologically active substances such as proteins, enzymes, and drugs [99]. The main substance loading strategies for RBCs are intra‐erythrocyte loading and erythrocyte surface adhesion, of which the former is more widely used. As early as 2005, researchers utilized electroporation to form transient pores on the surface of RBCs to load vitamin C and mannitol molecules into erythrocytes [100]. According to the characteristic of swelling and deformation of RBCs in hypotonic environment, through hypotonic dilution [101] and hypotonic dialysis [102], the RBCs absorb water and swell and open the hole on the surface of the cell membrane in hypotonic environment, and the delivered substance enters into the RBCs through this hole, and then the RBCs are placed in hypertonic solution to close the hole on the surface, realizing the encapsulation of the substance. In addition, low molecular weight chitosan NPs prepared by gel technology can be adsorbed on the membrane of erythrocytes, which is a potential vascular drug delivery pathway [103]. The process of drug release within the erythrocyte membrane depends mainly on the binding force of the drug to the erythrocyte membrane, as well as the influence of external factors such as temperature and pH. When RBCs reach a specific site, the erythrocyte membrane may fuse with the target cell membrane or enter the cell through receptor‐mediated endocytosis, thus releasing the drug. In addition, changes in intravascular temperature and pressure, or a decrease in pH in the TME can contribute to the gradual release of the drug [104].

RBCs have many advantages as carriers of NPs, for example, their long circulating half‐life increases the cycle of the substance in the body [105]; the surface of the erythrocyte membrane contains the glycoprotein CD47, which prevents them from being phagocytosed by macrophages, evading the immune system, and exhibiting a longer lifespan within the TME [94]. In addition, better biocompatibility and high loading capacity, which can improve the stability of NPs, are all advantages of erythrocyte carriers, which are widely used for the delivery of antitumor drugs [106]. However, RBCs lack tumor targeting and may undergo structural changes and loss of activity during loading of substances, thus losing their delivery function [107]. To address these shortcomings, erythrocyte membrane‐derived vesicles have been developed.

These vesicles are formed by rupturing erythrocytes in a hypotonic solution, releasing their contents, washing them repeatedly, and then extruding them several TMEs through a polycarbonate porous membrane to form vesicles of different sizes [108]. It is more stable than the simple erythrocyte membrane, with a slow release of drugs and a wider range of applications. Many studies have been conducted to make antitumor drugs into NPs and encapsulate them into erythrocyte membrane‐derived vesicles, which have been experimentally demonstrated to have higher drug loading and particle size stability, better uptake by tumor cells, stronger cytotoxicity, and reduced side effects brought by the traditional drug delivery methods to a certain extent [109, 110]. Moreover, the targeting of erythrocytes can be enhanced by modifying their membranes. RBCs modified with lipophilic anti‐CD45 antibody can bind effectively to leukocytes, and RBCs modified with anti‐CD20 can effectively kill CD19+/CD20+/CD45+ human lymphoma cells [111].

Erythrocyte membrane mimetic NPs are rapidly developing in the field of targeted drug delivery and have good application prospects, but also have certain limitations and challenges. For example, the preparation process of erythrocyte membrane‐derived vesicles is relatively complicated, the production technology is not perfect, the stability of nanomedicines cannot be guaranteed, and the industrialized manufacturing of vesicles is still difficult. Furthermore, in terms of cell membrane source, it is more recommended to use an autologous source of erythrocytes to minimize the immune response to NPs, and to avoid potential allergic reactions in case of an allogeneic or xenogeneic source of cells [97]. With the continuous progress of nanotechnology, more efficient delivery tools will be developed in the future, and new approaches will be developed for the drug treatment of tumors.

#### 3.1.2 Cancer Cell Membrane‐Camouflaged Nanomedicines

Among the treatment methods for cancer, conventional radiotherapy, chemotherapy, and novel immunotherapy can achieve certain efficacy, but they are unable to specifically target tumor cells, and additional radiation and off‐target chemotherapeutic drugs will have unavoidable toxic side effects on the normal organism [112]. Therefore, delivering antitumor drugs to the tumor to precisely kill tumor cells without affecting the function of normal cells is one of the urgent problems to be solved. With the application of NPs in drug delivery systems, cancer cell membrane biomimetic NPs have been developed for delivering anticancer drugs to achieve precise treatment of tumors.

Cancer cells possess strong immune escape and homologous binding ability, using cancer cell membrane as a coating material for nano anticancer drugs can prolong the circulation TME of the drugs in the body, avoiding being cleared by the immune system and targeting the homotypic tumors [113]. As cells with unlimited replication and division potential, cancer cells are easier to culture in vitro with sufficient membrane sources. Compared with mature erythrocytes without nucleus, the extraction and purification of cell membranes from nucleated cells are more complicated. Cell membranes were obtained by mechanical squeezing, repeated freeze‐thawing, and hypotonic solution treatment, and then the nuclei and various intracellular biomolecules were removed by gradient centrifugation to obtain tumor cell membrane vesicles [114, 115]. Extrusion, ultrasound, and microfluidic techniques allow for the fusion of tumor cell membrane vesicles with the NPs, and gentle manipulation at low temperatures maintains the bioactivity of the resulting membranes to a greater extent [116]. When the biomimetic NPs reach the acidic TME with blood circulation, pH‐sensitive materials can be used to make the drug vesicles release the drug in a low pH environment. In addition, photosensitive and thermosensitive materials can also induce vesicle structure change and drug release, thus improving drug targeting and release precision, reducing drug side effects, and enhancing therapeutic efficacy [117, 118].

Similar to erythrocytes, a variety of tumor cells overexpress CD47 molecules on their surface, thereby evading phagocytosis by macrophages [119]. Moreover, N‐calmodulin, galectin‐3 and epithelial cell adhesion molecules on the surface of tumor cells give them homologous adhesion properties, and NPs encapsulated in the tumor cell membranes have better tumor targeting properties and are more likely to adhere to homotypic tumor cells, which facilitates the uptake and precise release of drugs [3]. In addition, antigens carried on the membrane surface of cancer cells can be specifically recognized by DCs and activate antigen‐specific T cells, thereby generating an antitumor response against the primary tumor cells in vivo [120]. Experiments have shown that biomimetic NPs encapsulating chemotherapeutic drugs with 4T1 breast cancer cell membranes have longer drug half‐life and higher tumor targeting than simple NPs, and exhibit excellent antitumor and anti‐metastasis efficacy in tumor models [121]. To improve the efficacy of tumor photothermal therapy, researchers developed bismuth (Bi) metal NPs encapsulated in the membranes of colon cancer CT26 cells, which had 1.2 TMEs the absorption rate of ordinary NPs in CT26 cells, longer half‐life and better tumor tropism. Laser irradiation induces the death of almost all tumor cells that have ingested NPs, resulting in a significant reduction in tumor size [122]. Leveraging their inherent homotargeted and immune‐evasive properties, cancer cell membrane‐camouflaged NPs infiltrate tumor tissues like precision‐engineered Trojan horses.

Despite the significant advantages of tumor cell membrane camouflaged biomimetic nanomedicines in the precise treatment of cancer, there are some drawbacks and limitations. The extraction and purification steps of cancer cell membranes are more complicated, making it difficult to achieve large‐scale production, and can be affected by tumor types, culture conditions, and several other factors, resulting in poor stability and consistency of the preparation [123]. Although tumor cell membrane camouflage reduces the possibility of recognition by the immune system to a certain extent, the tumor cells still contain certain antigens capable of being recognized, and thus cleared by the immune system and reduce the therapeutic effect. Therefore, further research and more advanced technologies are needed to overcome these shortcomings in the future and provide a powerful tool for antitumor therapy.

#### 3.1.3 Immune Cell Membrane‐Camouflaged Nanomedicines

Immune cells are an important part of the TME and a powerful weapon to kill tumor cells, but the formation of an immunosuppressive TME results in the inability of some cells to perform their antitumor functions effectively. Based on the tumor‐targeting properties of certain immune cells, the development of immune cell membrane‐encapsulated nanomedicines can transport therapeutic drugs to the tumor in a targeted manner, increasing the accumulation of the drugs in the tumor cells and improving the therapeutic efficacy [98]. Macrophages, DCs, NK cells, lymphocytes, etc. have been fabricated into biomimetic NPs, which inherit the different properties of their respective cells and play unique and diverse roles in the delivery of antitumor drugs [124].

M1 macrophages have powerful antigen‐presenting, pro‐inflammatory, phagocytic capacity, and natural inflammatory targeting, and are chemotactic for both primary and metastatic tumors, therefore, they are widely used as drug carriers to penetrate deep into tumor regions that are poorly perfused by conventional drugs, which facilitates the treatment of cancers that are resistant to radiotherapy and chemotherapy [98]. A large number of studies have focused on macrophage membrane‐encapsulated NPs as drug delivery vehicles, where macrophage membrane masking prevents them from being recognized and cleared by the mononuclear phagocytosis system, thus prolonging the circulation TME of the drug in the body and allowing for a gradual release of the drug in vivo [125, 126]. In a mouse model of metastatic breast cancer, cytotoxic NPs encapsulated with M1 macrophage membranes showed better antitumor metastasis effects than unencapsulated NPs in lymph nodes and lungs, which may be attributed to the fact that macrophage membrane‐encapsulated NPs can improve their tumor‐targeting ability through various protein receptors on the membranes and facilitate adhesion to tumor cells and endocytosis of NPs [127]. In addition, a hybrid membrane was prepared by fusing macrophages and 4T1 breast cancer cells, which successfully realized drug targeting delivery to lung metastases of breast cancer. This hybrid cell membrane retains the specific biological properties and protein markers of macrophages and cancer cells, and the homotypic targeting ability of cancer cells and the metastatic targeting ability of macrophages are simultaneously enhanced, which develops a promising therapeutic approach for the treatment of metastatic breast cancer [128]. In addition, integrin α4 and Mac‐1 proteins on the surface of macrophage membranes can bind to the corresponding receptors on cerebrovascular endothelial cells, such as vascular cell adhesion molecule (VCAM)‐1 and intercellular cell adhesion molecule (ICAM)‐1, which can facilitate the crossing of macrophage membrane‐modified NPs through the blood‐brain barrier (BBB) by reducing the expression of tight junction‐associated proteins, which is beneficial for the treatment of glioblastoma [129]. However, macrophage biomimetic NPs also have the disadvantage that the SIRPα on their surface can bind to CD47 on the surface of tumor cells, which causes the tumor to send a “don't‐eat‐me” signal to the macrophage and prevents it from being phagocytosed [119]. In addition, macrophages in the TME are susceptible to differentiation into an inflammation‐suppressive M2 phenotype under the action of various cytokines secreted by tumor cells, which promotes tumor progression.

T lymphocytes are the major force of antitumor immunity, and the TCRs on their surface can specifically recognize and bind tumor antigens, as well as numerous adhesion molecules that bind with high affinity to tumor‐associated antigens, enabling them to accurately target cancer cells. Therefore, T cell membrane is a good candidate for making biomimetic nanodrug particles, which can inherit the functions of T cells to actively target tumor cells and mediate immune responses, which is conducive to the precise treatment of cancer [130]. Anticancer chemotherapeutic drugs are encapsulated in melanoma‐specific anti‐gp100/HLA‐A2 TCR expressing T cell membranes to make biomimetic NPs for the treatment of malignant melanoma, which were experimentally proven to have good biocompatibility and stability, and were able to achieve long‐lasting release of the drugs, and higher TCR concentration results in higher cellular uptake rate and cytotoxicity of the NPs [131]. In addition, the biomimetic NPs made of paclitaxel wrapped in CTL membranes can not only avoid phagocytosis by macrophages, but also use the adhesion molecule LFA‐1 on the cell membrane to actively target tumor cells, which is conducive to the targeted release of drugs. Combined with local low‐dose irradiation, tumor‐specific T cells were recruited to the tumor site, effectively inhibiting the progression of gastric cancer [132]. Compared to the expensive and complex in vitro processing required for adoptive T cell therapy, T cell membrane biomimetic NPs are relatively easy to prepare and manipulate and are more widely applicable, allowing for long‐term circulation of the drug in the bloodstream, greater targeting and specificity, and fewer side effects, minimizing unwanted immune responses.

Similar to the preparation process of cancer cell membrane‐encapsulated NPs, immune cell membrane‐encapsulated NPs need to go through the extraction of the outer membrane, isolation of the membrane vesicles, and fusion of the nanovesicles with the NP core by co‐extrusion, ultrasonication, and electroporation [116]. Currently, large‐scale production and storage remain one of the major obstacles to the clinical application of biomimetic nanomedicines. Extraction of cell membranes, extrusion, and fusion processes may disrupt cell surface molecules and lead to inactivation of functional proteins. MHC molecules on immune cell membranes, as well as antigens generated by contamination during the preparation process, pose additional immunogenicity problems. In order to solve these problems, the optimization and development of preparation techniques should be further advanced in the future.

### 3.2 Biomimetic Nanomedicines Based on Extracellular Vesicles (EVs)

Beyond immune cell‐derived membranes, naturally secreted extracellular vesicles provide another biologically relevant platform for biomimetic delivery. Extracellular vesicles (EVs) represent a sophisticated biological delivery paradigm wherein cells secrete nanoscale lipid assemblies through evolutionarily conserved pathways. These membrane‐bound entities, primarily classified as exosomes and microvesicles, originate via distinct biogenetic processes [133, 134]. Exosomes derive from endosomal maturation pathways involving multivesicular body formation, while microvesicles emerge through calcium‐dependent plasma membrane remodeling. Their molecular architecture preserves parental cell surface proteomes and lipidic signatures, facilitating selective packaging of bioactive molecules, including non‐coding RNAs and signaling proteins [135]. Isolation methodologies leverage differential biophysical properties, with ultracentrifugation serving as the foundational approach complemented increasingly by size‐exclusion chromatography and immunoaffinity techniques targeting conserved surface markers [136, 137]. Cargo loading exploits intrinsic membrane properties through passive diffusion or transient pore formation strategies, maintaining vesicle integrity while encapsulating therapeutic payloads. This endogenous transport system inherently overcomes biological barriers through native ligand‐receptor recognition and membrane fusion capabilities unattainable by synthetic nanocarriers [138].

Functional diversification arises from vesicular ontogeny. Exosomes mediate targeted intercellular communication via surface protein complexes that orchestrate tissue‐specific homing, particularly enabling transcellular barrier penetration [139]. Their molecular payloads further facilitate epigenetic reprogramming of recipient cells, establishing their utility as natural gene delivery vectors [140]. Microvesicles exhibit specialized signaling through externalized phospholipid‐mediated cellular uptake and integrin‐directed anchoring, efficiently transferring macromolecular complexes [141]. Both subtypes confer critical therapeutic advantages: biomimetic stealth properties circumvent immune surveillance, endogenous targeting ligands ensure precision delivery, and phospholipid bilayers protect labile cargos from degradation [142]. Nevertheless, clinical translation confronts multifaceted barriers. Significant vesicle heterogeneity persists across cellular sources and culture conditions, complicating therapeutic standardization. Scalable production remains constrained by donor cell productivity limitations and technically intensive purification workflows requiring manufacturing compliance [143]. Currently, the large‐scale production of extracellular vesicles poses a significant challenge for clinical applications. Researchers have employed methods such as genetic engineering, bio‐orthogonal click chemistry, microfluidics, and microarray chips to create highly efficient, controllable, and mass‐producible therapeutic extracellular vesicles [144, 145]. This advancement offers a potential solution for personalized precision cancer treatment.

### 3.3 Virus‐Mimicking Nanomedicine

Viruses exemplify evolutionarily refined nanocarriers that achieve targeted cellular delivery through ligand‐receptor mediated tropism while evading host immunity. This innate biological intelligence has inspired engineered viral vectors (e.g., adenoviral, lentiviral systems) demonstrating high transfection efficacy yet limited by inherent pathogenicity and immunogenicity [146, 147]. Contemporary virus‐mimetic NPs resolve this dichotomy through rational biodesign: they preserve structural proteins governing host cell recognition and membrane fusion mechanisms while eliminating genetic components responsible for replication [148]. These pathogenically attenuated platforms precisely replicate viral entry competencies, enabling efficient delivery of nucleic acid therapeutics to specific cell populations. This strategic emulation of natural viral architectures balances biological targeting precision with clinical safety, meeting the requirements for oncological and genetic disorder interventions [149, 150].

Virus‐like particles (VLPs) represent advanced biomimetic systems self‐assembled from viral structural proteins, categorized by envelope presence [151]. Non‐enveloped VLPs leverage geometrically precise capsid cavities for therapeutic encapsulation, while enveloped variants integrate glycoprotein‐lipid membrane complexes that simultaneously mask immunogenic epitopes and direct cellular targeting [152]. Their functional sophistication enables innovative payload integration: capsid cavity loading exploits quaternary structure specificity for organotropic delivery (e.g., hepatocyte‐homing adenoviral dodecahedrons enhancing doxorubicin efficacy in hepatic carcinoma) [153], whereas protein‐conjugation strategies harness natural viral affinities (e.g., rotavirus VP6‐mediated hepatocellular targeting) [154]. However, VLP translation faces dichotomous challenges, inherent immunogenicity that benefits vaccine development but risks inflammatory complications in therapeutic delivery, alongside manufacturing complexities in achieving structural fidelity at scale [155]. Addressing these requires orthogonal solutions: epitope‐editing methodologies to fine‐tune immune activation, microbial biofactories for scalable production, and continuous‐flow bioprocessing to standardize self‐assembly kinetics [156]. This multipronged advancement strategy positions VLPs to transcend current limitations as next‐generation precision nanotherapeutics.

### 3.4 Nanozymes

Nanozymes are a class of functional nanomaterials that exhibit enzyme‐like catalytic activities, which can be categorized into hydrolases, oxidoreductases, transferases, and others based on their functions. Compared to natural enzymes, nanozymes offer superior stability, cost‐effectiveness, and scalability for mass production, along with unique physicochemical properties conferred by their nanomaterial composition. These advantages have garnered significant attention in recent years, particularly in the development of novel anticancer strategies [157]. Nanozymes with peroxidase‐ and oxidase‐like properties can kill tumor cells by elevating intracellular ROS levels, while those with superoxide dismutase (SOD) activity can indirectly induce tumor cell death by ameliorating hypoxia in the TME [158]. Under hypoxic conditions, cancer cells can generate a large amount of hydrogen peroxide (H_2_O_2_). By utilizing the catalase‐like activity of manganese dioxide (MnO_2_) NPs, intravenous injection of these nanozymes into tumors can increase the oxygen content in the TME, thereby enhancing tumor‐specific PDT and significantly inhibiting tumor growth [159]. RePd@OMVsPD‐L1 nb is an intelligent biomimetic immune organelle that exhibits peroxidase and glutathione peroxidase activities by combining nanozymes and bacterial outer membrane vesicles (OMVs) [160]. It not only generates ROS to kill tumor cells, but also utilizes outer membrane vesicles to activate the immune system and target PD‐L1, thereby inhibiting the growth of bladder cancer cells. A variety of methods such as electrochemical deposition, co‐precipitation, and chemical reduction can be used to prepare many types of nanozymes with different functions [161]. With the development of nanotechnology, its application in the medical field is expanding, but the biosafety of nanozymes is noteworthy, and its potential cytotoxicity and pharmacokinetics are still unproven. On the other hand, the targeting efficacy, specificity and in vivo activity of nanozymes are still unclear, which may affect biosafety [162]. Therefore, more detailed studies and analyses of nanozymes are needed for their safe use in clinical therapy in the future.

### 3.5 Bacteria‐Camouflaged Nanomedicines

Bacterial biomimetic nanomedicines represent an emerging class of oncological therapeutics that mimic or harness the unique biological properties of bacteria and their components to construct nanoscale drug delivery systems, offering novel strategies, and powerful tools for cancer treatment [163]. Genetically engineered live bacteria can serve as delivery vehicles for nanomedicines, leveraging the inherent chemotaxis of anaerobic bacteria toward the hypoxic TME to achieve active accumulation within tumors. Alternatively, coating synthetic NPs with extracted bacterial outer membranes creates a “camouflage” that endows the NPs with certain surface proteins and antigenic properties of the bacterial membrane [164]. Liu et al. designed biomimetic NPs covered with Salmonella membrane (SM‐AuNR), and the specific proteins on the bacterial membrane surface can target tumor cells. The NPs have a rod‐shaped morphology and can effectively penetrate the mucosal barrier and tumor stroma, promoting particle internalization. In addition, NPs loaded with chemotherapy drugs Dox@SM‐AuNRs combines photothermal therapy (PTT) and chemotherapy, showing significant antitumor effects in a mouse in situ colorectal cancer model [165].

Despite the promising prospects, the clinical translation of bacterial biomimetic nanomedicines faces numerous challenges. Their immunogenicity and potential toxicity must be effectively controlled, while the processes for extracting and purifying bacterial components and fusing them with NPs are complex, making standardized industrial production difficult [166].

## 4 Biomimetic Nanomedicines Targeting the TME

Biomimetic nanomedicines possess multiple unique properties that enable them to actively reprogram the tumor microenvironment through diverse mechanisms. This section explores how they directly interact with the tumor microenvironment at cellular, metabolic, and physical levels to alter its immunosuppressive state, thereby serving as powerful tools for tumor immunotherapy.

### 4.1 Immune Cell Reprogramming

**FIGURE 5:**
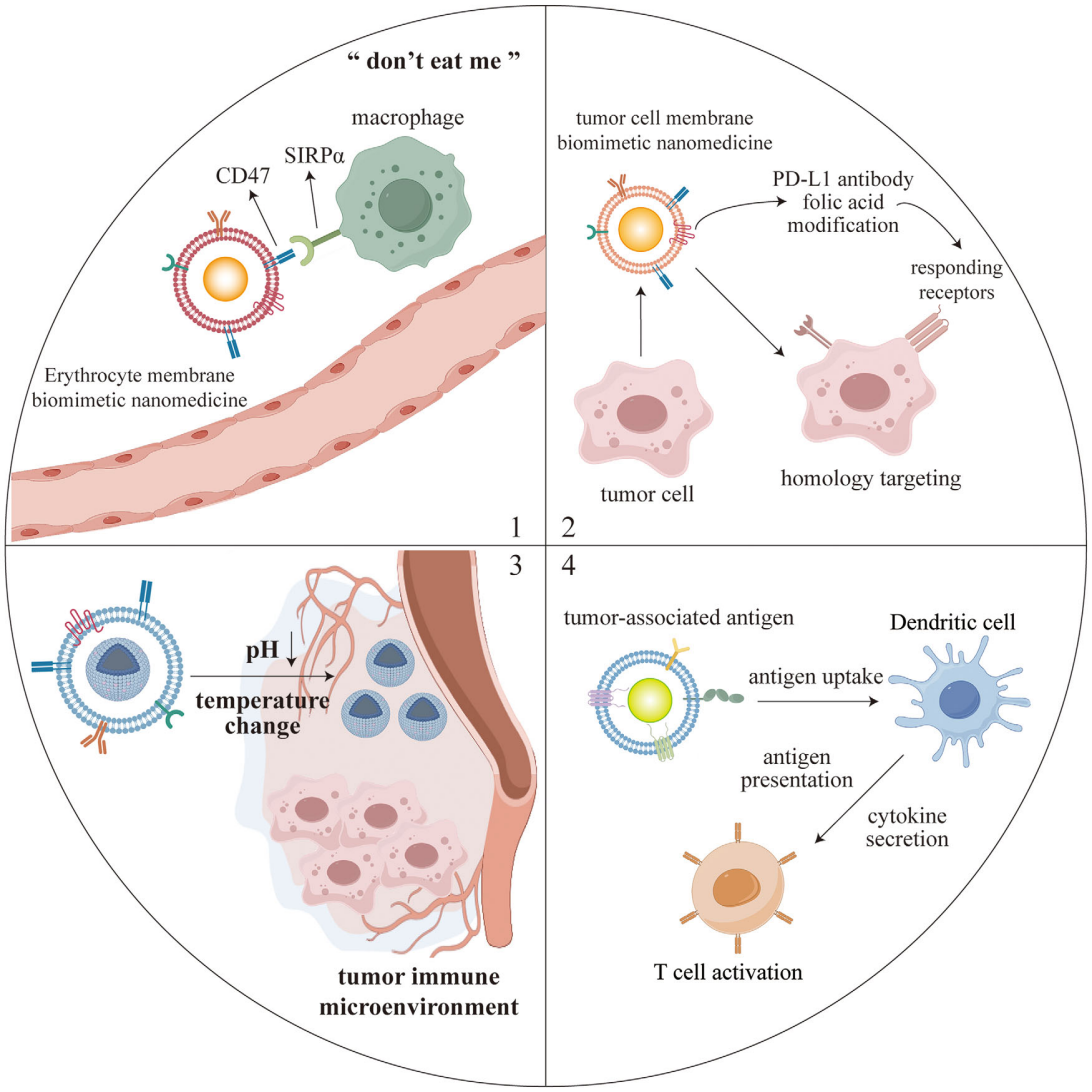
Mechanisms of biomimetic nanomedicine targeting of TME. (1) Circulatory escape; (2) tumor targeting; (3) microenvironmental response; (4) immune activation.

Immune cell reprogramming refers to the modification of immune cell function or characteristics through techniques such as gene editing, pharmacological intervention, or cytokine treatment, enabling them to acquire novel capabilities in antitumor immunity, anti‐infection responses, or immunomodulation. Changes in the morphology, structure, and metabolic reprogramming of immune cells can profoundly influence their functionality, playing a pivotal role in various pathophysiological processes, including cancer, autoimmune diseases, and aging [36]. Tumor cells typically exhibit heightened metabolic activity, preferentially consuming nutrients such as glucose, amino acids, and fatty acids, which deprives immune cells of essential resources and compromises their functionality. Additionally, hypoxia and lactate accumulation further impair the metabolism and function of T cells and NK cells, promoting immune evasion of tumor cells [167]. Leveraging biomimetic nanomedicines to induce immune cell reprogramming and modulate their functional states holds significant promise for enhancing antitumor effects, representing a critical strategy in advancing cancer immunotherapy. Figure 5 demonstrates the mechanism by which biomimetic nanomedicines target TME.

#### 4.1.1 Immunosuppressive Cell Reprogramming

Immunosuppressive cells in TME can help tumors evade the host immune system, therefore, targeting these cells is an important strategy to restore antitumor responses. Some studies have shown that biomimetic NPs can target immunosuppressive cells in the TME while delivering anticancer drugs. By utilizing immune‐activating factors or immune checkpoint inhibitors, these NPs can induce cellular reprogramming, suppress their immunosuppressive effects and promote immune system to attack tumors [168].

Researchers developed a biomimetic nanomedicine loaded with gemcitabine, which is coated with bioengineered cancer cell membranes featuring peptides that target M2‐type macrophages and TAAs in pancreatic cancer cells. This drug reprogrammed macrophage phenotype, decreased the M2 type proportion while increasing the M1 type, which could induce more tumor cells apoptosis and improved the efficacy of chemotherapy [169]. In addition, lanthanum‐nickel oxide (LNO) nanozymes with phosphatase‐like activity can enhance the antitumor effect in tumor‐bearing mice by depleting adenosine triphosphate (ATP)‐induced autophagy in TAMs and promoting macrophage polarization from M2 to M1 phenotype. Encapsulation of myeloid cell membranes on the surface of LNO reduces its clearance by the reticuloendothelial system and increases its accumulation in the TME, resulting in a significant enhancement of antitumor immunity [170].

Besides, Tregs infiltration in the TME is closely related to cancer progression. Latexin (LXN) is a tumor suppressor, and LXN in macrophage‐derived exosomes can inhibit the differentiation of CD4+T cells into Treg cells, thus exerting an inhibitory effect on tumor growth. Based on this, researchers developed biomimetic NPs containing LXN proteins and camouflaged with macrophage membranes, which significantly reduced the infiltration of Tregs into tumor tissues when injected into tumor‐bearing mice [171]. Given that Tregs in the TME are highly CD25‐expressing, anti‐CD25 antibodies have been developed to deplete immunosuppressive Tregs to promote the efficacy of cancer immunotherapy [172]. However, CD25 is also expressed on effector T cells, and conventional CD25 antibodies block their activation signals while suppressing antitumor activity. This challenge can be addressed through biomimetic nanomedicine platforms engineered to deliver Treg‐specific CD25‐targeting antibodies, which selectively deplete Tregs without compromising effector T cell populations, thereby enabling more precise TME remodeling.

#### 4.1.2 Effector Cell Reprogramming

While reprogramming immunosuppressive cells alleviates the inhibitory pressure of the TME, activating effector cells further amplifies the offensive capacity of antitumor immunity. These two complementary strategies collectively restore the antitumor function.

**FIGURE 6:**
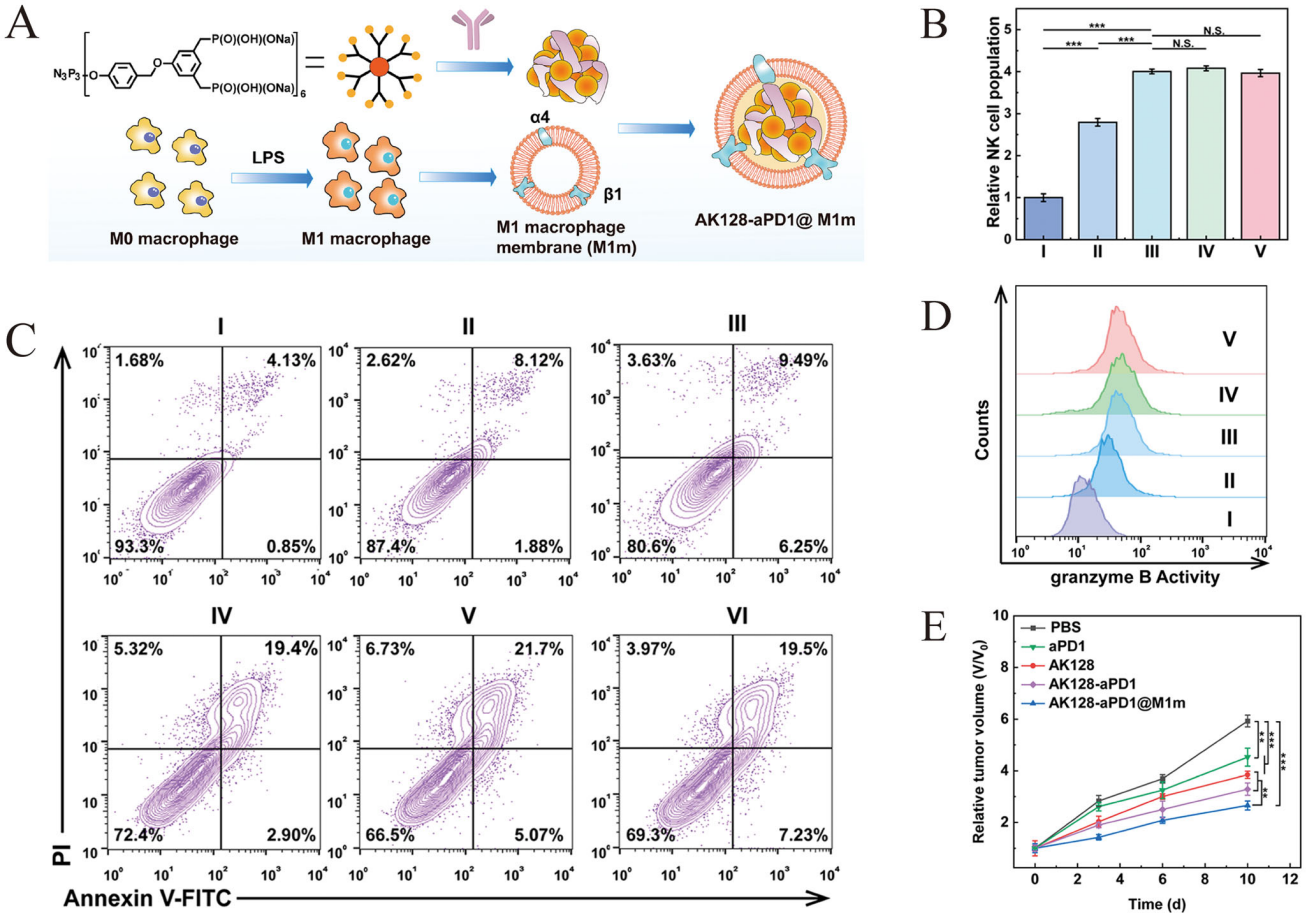
(A) The synthesis process of biomimetic nanomedicine. (B) Representative flow cytometry quantification of NK cell populations after treating PBMCs with different formulations. (C) Flow cytometry analysis of apoptosis and necrosis of C6 cells after 12 h of different treatments. (D) Fluorescence intensity histograms of granzyme B after treating PBMCs with different formulations. (E) The relative tumor volume of in situ gliomas in mice after different treatments. For (B and D): I, PBS; II, IL‐2; III, IL‐2 + AK128; IV, IL2 + AK128‐aPD1; V, IL‐2 + AK128‐aPD1@M1m. For (C): I, PBS; II, IL‐2 + PBS; III, IL‐2 + aPD1; IV, IL‐2 + AK128; V, IL‐2 + AK128‐aPD1; and VI, IL‐2 + AK128‐aPD1@M1m. Reprinted with permission from Ref. [173]. Copyright (2024), American Chemical Society.

The M1 macrophage membrane‐camouflaged phosphorus dendrimer (AK128)/programmed cell death protein 1 antibody (aPD‐1) nanocomplex is capable of penetrating the BBB, delivering AK128 and aPD‐1 with intrinsic immunomodulatory activity to gliomas. Experimental evidence demonstrates that AK128 promotes NK cell proliferation, and aPD‐1 restores the tumor‐killing activity of CTLs and NK cells through ICB, thereby promoting tumor cell apoptosis and improving the efficacy of glioma immunotherapy [173]. Figure 6A shows the synthesis process of biomimetic nanomedicine. Figure 6B,D indicates that AK128 can promote NK cell proliferation, and treatment with AK128 resulted in higher expression of granzyme B compared to treatment with PBS. Figure 6C shows that the apoptosis and necrosis rates of C6 cells treated with aPD‐1 or AK128 (in the presence of IL‐2) were significantly higher than those in the PBS group. The efficacy of biomimetic nanomedicine in inhibiting tumor growth has been further validated in mouse models (Figure 6E).

**FIGURE 7:**
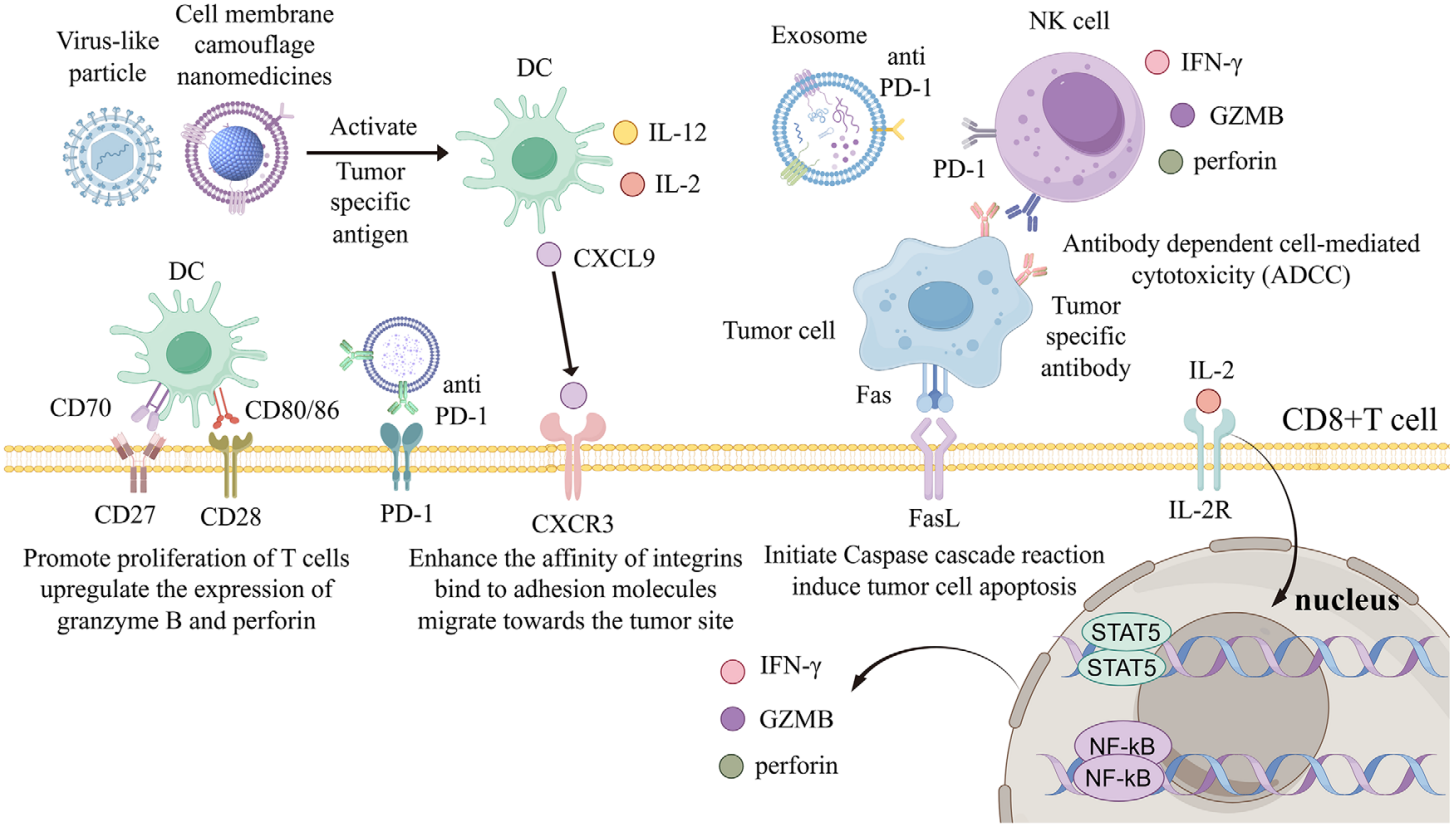
Different types of biomimetic nanomedicines activate CD8+T cells through various mechanisms.

In addition, there have been numerous studies exploring how biomimetic nanomedicines activate CD8+T cells through various mechanisms (Figure 7). Tumor cell membrane‐coated biomimetic NPs can be loaded with tumor‐specific antigens (TSAs) to activate DCs, prompting the secretion of cytokines and chemokines. This activation, mediated through cell surface receptors, promotes the differentiation of T cells into CD8+ T cell and upregulates transcription factors that enhance the expression of perforin and granzymes (GZMB). Additionally, anti‐PD‐1 antibody‐modified exosomes can block the PD‐1/PD‐L1 signaling pathway, thereby preventing the loss of antitumor function in T cells and NK cells. Furthermore, tumor‐specific antibodies can mediate antibody‐dependent cellular cytotoxicity (ADCC) by NK cells, enhancing antitumor immunity from a new perspective.

### 4.2 Improvement of the Hypoxic Microenvironment

Hypoxia in the TME is a common feature of solid tumors, mainly due to the disordered vascular structure of tumors and uneven blood flow, resulting in insufficient oxygen supply [174]. Hypoxia not only promotes tumor progression but also reduces the effects of radiotherapy, chemotherapy, and immunotherapy. Utilizing biomimetic nanomedicine to increase oxygen supply, reduce oxygen consumption, and improve the hypoxic environment is a novel strategy to eliminate tumors [175]. Various oxygen supplementation strategies have been developed, for example, hemoglobin and oxygen nanocarriers can delivering oxygen to tumor to increase oxygen concentration [176]. A biomimetic nanomedicine system was developed by encapsulating C6 cell membranes with doxorubicin and MnO_2_. This system not only decomposes H_2_O_2_ to produce oxygen to alleviate hypoxia, but also increases ROS induced oxidative stress to kill cancer cells. Meanwhile, doxorubicin is only released in the acidic environment, which helps to reduce the toxicity and side effects of chemotherapy drugs [177]. Hemoglobin in a biomimetic nano RBC system can deliver oxygen, thereby improving hypoxia and reducing M2 type TAMs recruitment, meanwhile, it can specifically target and kill TAMs through surface receptor CD163, improving the immunosuppressive state of TME, and enhancing the antitumor effect of CTLs [178]. Restoration of immune cell activity and functionality following hypoxia reversal augments therapeutic efficacy of cancer immunotherapy.

### 4.3 Regulation of the Acidic Microenvironment

**FIGURE 8:**
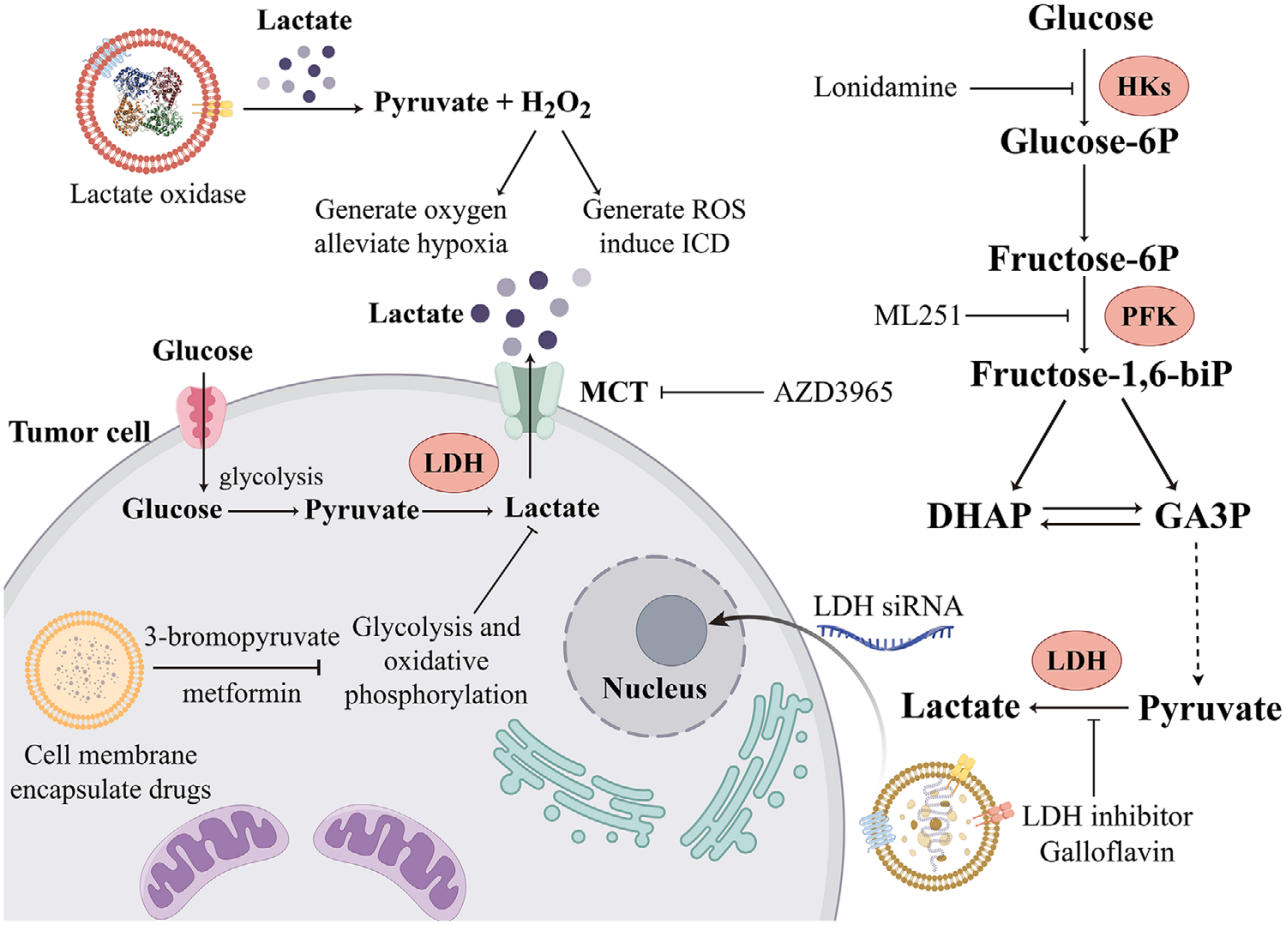
Application of biomimetic nanomedicine in improving the acidic environment of tumors.

Tumor cells have an abnormally active metabolism, and driven by their high energy demand, they usually rely on the glycolytic pathway to generate energy, which leads to the accumulation of lactate, causing acidification of the TME and further promoting tumor aggressiveness and drug resistance [8]. Therefore, regulating the acidity of the TME and inhibiting lactate accumulation are important in cancer therapy. Recent studies have utilized M1 macrophage membranes to encapsulate metformin and 3‐bromopyruvate, creating biomimetic NPs that can be selectively taken up by 4T1 breast cancer cells and induce apoptosis by inhibiting oxidative phosphorylation and glycolysis in cancer cells, effectively delaying tumor growth. Concurrently, the disruption of cancer cell energy metabolism significantly reduces lactate production, thereby ameliorating the acidic TME [179]. In addition, Fang et al. developed a novel immune nanomodulator [180]. Fluorocarbon chain‐modified mesoporous silica coated with liposomes can not only adsorb oxygen and improve the hypoxic state of the TME, but also consume H+ in the environment and prevent tumor cells from glycolysis to produce lactate, thereby preventing macrophages from polarization towards the M2 phenotype, and enhancing antitumor effects. Figure 8 summarizes the application of biomimetic nanomedicine in improving the acidic tumor microenvironment. Lactate oxidase can be incorporated into biomimetic NPs to catalyze lactate into pyruvate and hydrogen peroxide; the latter not only supplies oxygen but also acts as ROS to induce ICD in tumor cells. Meanwhile, nanoparticle systems coated with tumor cell membranes can be used to deliver drugs that inhibit glycolysis and oxidative phosphorylation into tumor cells, thereby reducing lactate production. Targeting key glycolytic enzymes and monocarboxylate transporters (MCTs), such biomimetic NPs enable efficient and specific drug delivery. Additionally, they can deliver small interfering RNA (siRNA) into tumor cells to silence the lactate dehydrogenase (LDH) gene, suppressing LDH expression at the genetic level.

### 4.4 Vascular Normalization

The vasculature of malignant tumors is characterized by high permeability and poor tissue perfusion, which creates hypoxia and acidic TME, promoting tumor proliferation, invasion, and metastasis, as well as impeding the successful entry of antitumor drugs into the tumor [181]. Normalization of tumor vessels is an important concept in tumor therapy research, by changing the structure and function of tumor vasculature to restore the state of normal vasculature, thus improving the TME, enhancing drug delivery, and improving the therapeutic efficacy [182]. Various strategies have been developed to induce normalization of tumor vasculature, such as inhibiting angiogenic factors, destroying abnormal blood vessels, and regulating endothelial cells and ECM [183]. The preparation of anti‐angiogenic drugs such as Bevacizumab [184], sorafenib [185], and rapamycin [186] into NPs can enhance drug activity and reduce the side effects of systemic administration. Additionally, the biomimetic NPs camouflaged by RBC membranes carry the endothelin A receptor antagonist BQ123, which can selectively dilate tumor blood vessels without affecting other blood vessels in the body, thereby promoting the accumulation of nanomedicines in tumor tissues [187]. The erythrocyte membrane‐encapsulated biomimetic NPs carry L‐arginine and photosensitizers, which can simulate the activity of nitric oxide synthase, achieve local NO release, inhibit the activation of platelets in tumor blood vessels, thereby enhancing vascular permeability and increasing the aggregation of chemotherapy drugs and biomimetic nanomedicines in tumors [188]. However, most of these studies are still in the experimental stage, and the drawbacks, resistance, and side effects of long‐term use are still undiscovered. Further studies are needed to confirm the feasibility of these therapies [189].

### 4.5 Modulation of ECM Enhances Immune Cell Infiltration

Tumor ECM is a highly dynamic network that is regulated by MMPs, cancer‐associated fibroblasts (CAFs), immune cells, and tumor cells themselves [190]. The dense ECM surrounding tumor cells acts as a barrier to drug delivery and fosters the formation of an immunosuppressive microenvironment. Consequently, disrupting and remodeling the tumor ECM has become a hot topic in cancer therapy [191]. With the rapid development of nanomedicine delivery systems, the use of NPs to precisely deliver substances that remodel the tumor ECM to the tumor region represents a novel therapy for the treatment of tumors.

**FIGURE 9:**
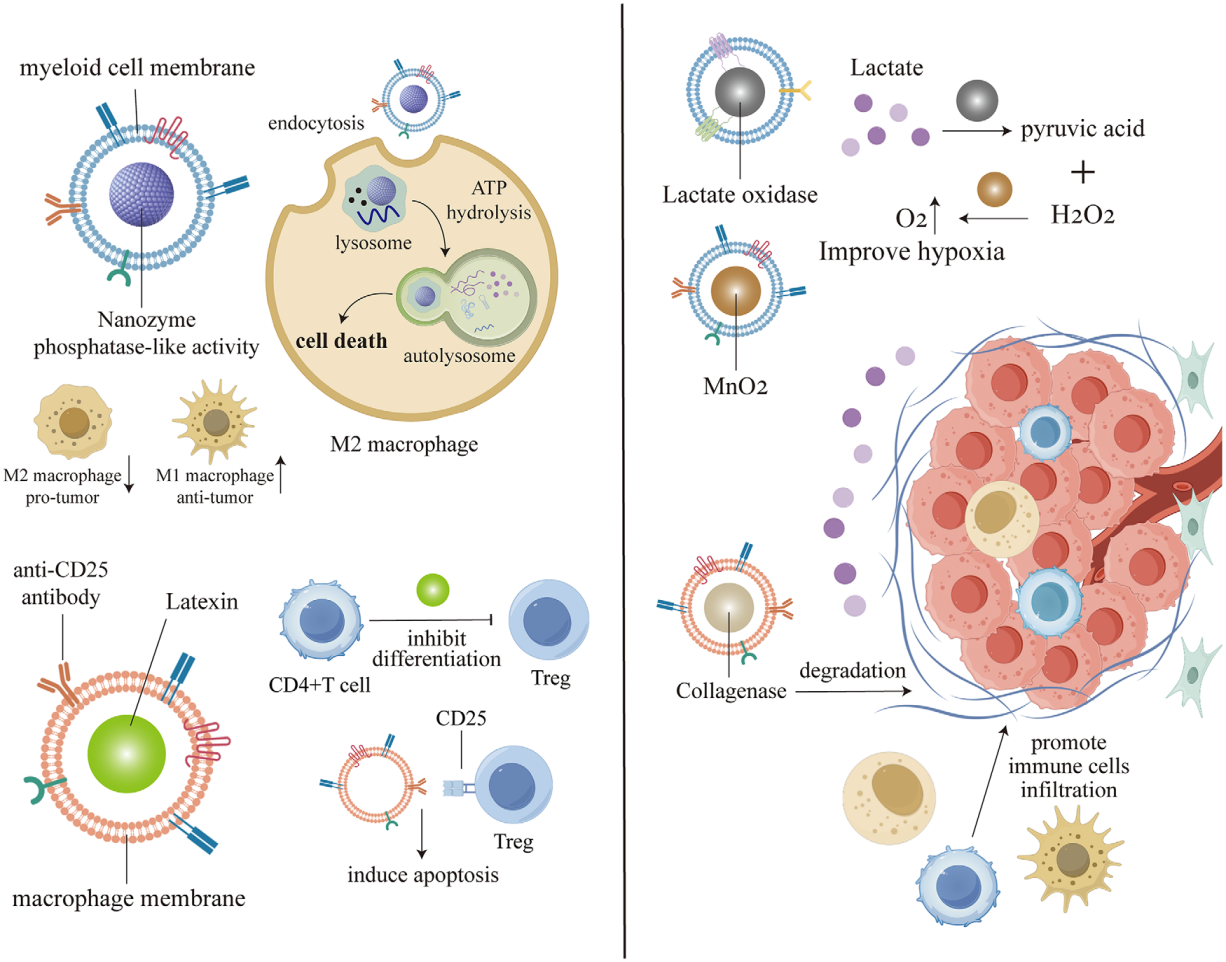
The mechanisms of biomimetic nanomedicine reprogramming TME: immune cell reprogramming; microenvironmental modulation; extracellular matrix barrier breakthrough.

Researchers constructed a “nanozyme capsule” encapsulated with collagenase and the chemotherapeutic drug doxorubicin to enhance tumor penetration of the drugs by hydrolyzing collagen in the tumor ECM. This capsule not only effectively protects the enzyme activity but also can be degraded in the acidic TME, releasing collagenase and chemotherapeutic drugs, and decreasing the density of the ECM, thereby increasing the aggregation and infiltration of drugs and immune cells in the tumor [192]. CAFs regulate the TME and ECM by secreting a variety of growth factors, which have been shown to promote tumor cell proliferation, invasion, and neovascularization. Therefore, elimination of CAF or inhibition of its function is beneficial for remodeling the ECM and limiting tumor progression [193]. A nanoparticle system was synthesized by chemically conjugating human relaxin‐2 with superparamagnetic iron oxide nanoparticle (SPION), which delays tumor growth by inhibiting TGF‐β‐induced CAFs differentiation and collagen production, thereby enhancing the efficacy of gemcitabine in pancreatic cancer [194]. Additionally, TGF‐β is an immunosuppressive factor, and its inhibition helps to ameliorate the immunosuppressive TME, promoting the antitumor activity of effector T cells. Figure 7 summarizes the synergistic mechanism of reprogramming TME with biomimetic nanomedicine (Figure 9).

## 5 Synergistic Applications in Immunotherapy

### 5.1 Induction of Immunogenic Cell Death (ICD)

ICD is a specific form of cell death characterized by the ability to trigger a specific immune response against antigens released by the dead cells during the dying process. ICD facilitates cancer treatment because it not only eliminates the tumor cells but also further activates the body's immune system and enhances the antitumor effects [195]. Immunogenic death of tumor cells produces a series of markers, such as calreticulin, which is exposed on the surface of tumor cells, and high mobility group box 1 (HMGB1) and ATP, which are released by the tumor cell into the extracellular space. They are also known as damage‐associated molecular patterns (DAMP), which can bind to pattern recognition receptors (PRRs) on the surface of DCs, thereby activating DCs and tumor‐specific CTLs, and enhancing antitumor efficacy [18]. Therefore, ICD is an important component of cancer immunotherapy and has a good therapeutic prospect, and how to induce ICD provides new ideas for the development of new drugs and clinical treatment. Research has shown that certain common chemotherapy drugs, such as oxaliplatin, 5‐fluorouracil, and doxorubicin can induce ICD while promoting tumor cell apoptosis, which is beneficial for improving the efficacy of chemotherapy. Making these drugs into biomimetic nanoparticles, targeting delivery to tumor areas, and precise release can alleviate severe adverse reactions associated with cytotoxic drugs to a certain extent [196, 197]. Recently, PTT and PDT have been extensively studied for their ability to induce ICD, and biomimetic nanomedicines can be combined with it to synergistically enhance the ICD effect and reverse the immunosuppressive TME [198].

**FIGURE 10:**
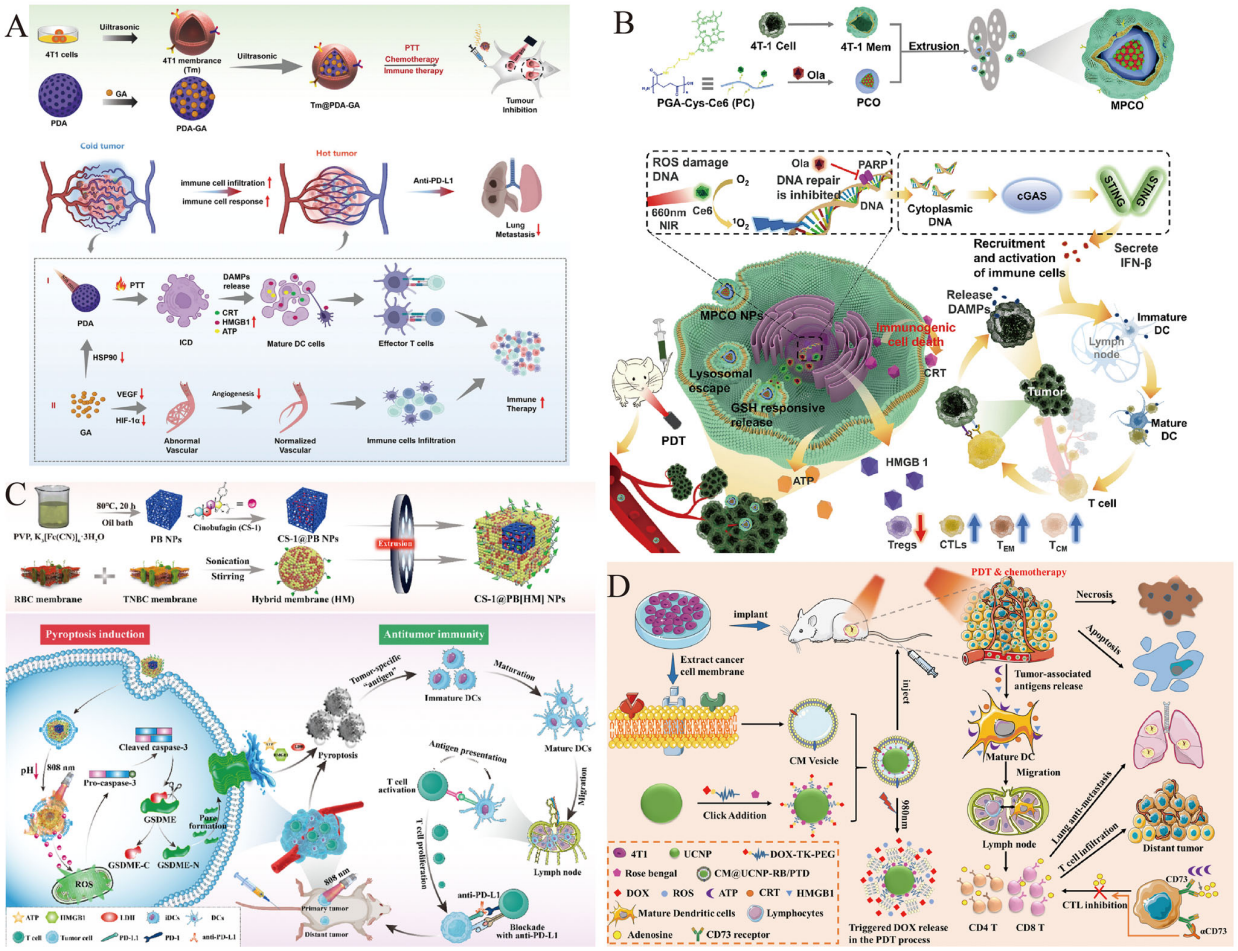
(A) Design and specific features of the drug delivery system Tm@PDA‐GA. I) PDA can induce PTT under 808 nm light irradiation, induce ICD, activate DCs, and stimulate cytotoxic T lymphocytes. II) GA can reduce the overexpression of HIF‐1α and VEGF, improve tumor vascular function, and enhance tumor immune infiltration. Reprinted with permission from Ref. [199]. Copyright (2024) Wiley. (B) Schematic diagram of the synergistic treatment of breast cancer based on a multifunctional biomimetic nanoplatform combining PDT and DNA damage repair inhibition. Reprinted with permission from Ref. [200]. Copyright (2023), Elsevier. (C) Schematic diagram of CS‐1@PB[HM] NP for comprehensive cancer treatment by inducing cell pyroptosis. Reprinted with permission from Ref. [201]. Copyright (2023), Elsevier. (D) Schematic diagram of the synthesis process of CM@UCNP‐RB/PTD, and its synergistic antitumor immunity in enhancing antitumor immunity in CD73‐blocking chemo‐photodynamic combination therapy. Reprinted with permission from Ref. [202]. Copyright (2021), Elsevier.

Lan et al. developed a biomimetic nanoparticle Tm@PDA‐GA, with 4T1 membrane encapsulation, reducing immunogenicity and promoting tumor cell uptake of the NPs. Polydopamine (PDA) serves as a drug carrier, inducing PTT and ICD under near‐infrared light irradiation to activate DCs. In addition, Tm@PDA‐GA can release glycyrrhizic acid (GA) in an acidic tumor microenvironment, inhibiting the expression of heat shock proteins (HSPs) and enhancing antitumor activity [199] (Figure 10A). Wu et al. utilized the membrane of mouse breast cancer 4T1 tumor cells to prepare 4T1Mem@PGA‐Ce6/Ola (MPCO) [200]. This biomimetic nanoparticle can homogeneously target tumor regions and release the photosensitizer Ce6 and olaparib. PDT is employed to generate ROS to damage tumor cell DNA and release DAMPs. Olaparib can inhibit DNA repair and activate the cyclic guanosine monophosphate‐adenosine monophosphate synthase (cGAS)‐stimulator of interferon gene (STING) pathway to produce immune mediators (Figure 10B). The fusion hybrid membrane of RBC and triple‐negative breast cancer (TNBC) cells is used to encapsulate a novel tumor‐targeted nanomedicine (CS‐1@PB[HM] NPs), enabling long‐term circulation and immune evasion in the body [201]. This nanomedicine has high drug loading efficiency and strong induction of cell pyroptosis. Laser irradiation of the tumor area can aggregate CS‐1@PB[HM] NPs, rapidly inducing GSDME‐dependent tumor cell pyroptosis, promoting the release of tumor antigens and DAMPs, and effectively enhancing T cell immune response (Figure 10C). The cancer cell membrane‐camouflaged NPs CM@UCNP‐RB/PTD utilize near‐infrared light to activate photosensitizers to generate ROS, inducing PDT while simultaneously combining doxorubicin to cause ICD and antitumor immunity. In addition, the NPs are blocked by CD73, inducing systemic cytotoxic T cell responses to prevent distant tumor metastasis (Figure 10D).

**FIGURE 11:**
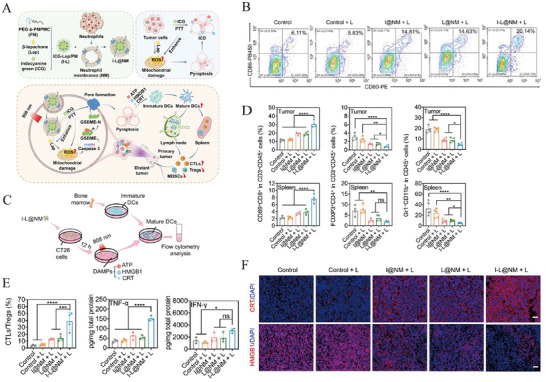
(A) Synthesis Concept of I‐L@NM and the Process of Pyroptosis‐Based ICD Induced by I‐L@NM. (B) The percentages of mature DCs following coculture with CT26 cells that had been pretreated in different ways. (C) Schematic of the in vitro procedure for stimulating DC maturation. (D) Percentage of CTLs, Tregs, and MDSCs in tumors and spleens. (E) The CTLs/Tregs ratios, and the levels of cytokines TNF‐α and IFN‐γ in the tumor. (F) Analysis of the distribution of CRT and HMGB1 in tumor slices by the immunofluorescence method. Reprinted with permission from Ref. [203]. Copyright (2023), American Chemical Society.

Neutrophil membranes were wrapped around polymers capable of spontaneously generating ROS to make biomimetic nanomedicines (I‐L@NM), which promoted ROS production under laser irradiation and photosensitizers, while enhancing gasdermin E‐mediated tumor cell pyroptosis, initiating a cascade reaction of ICD to effectively inhibit the growth and metastasis of tumor cells (Figure 11A). Coculture of CT26 cells with immature DCs showed that CT26 cells pretreated with I‐L@ NM + L significantly promoted the maturation of DCs (Figure 11B, C). The FCM analysis results show that I‐L@NM +In group L, the percentage of CTL cells was significantly higher than in other groups, and the number of Treg and MDSC cells was significantly reduced in this group (Figure 11D). It also elevated the ratio of CTLs to Tregs and cytokine TNF‐α and IFN‐γ content (Figure 11E). Staining tumor tissues with fluorescently labeled CRT and HMGB1 antibodies showed that I‐L@NM +After L treatment, there were significant changes in CRT and HMGB1 exposure in tumor tissue, and the ICD effect was very strong (Figure 11F) [203].

**FIGURE 12:**
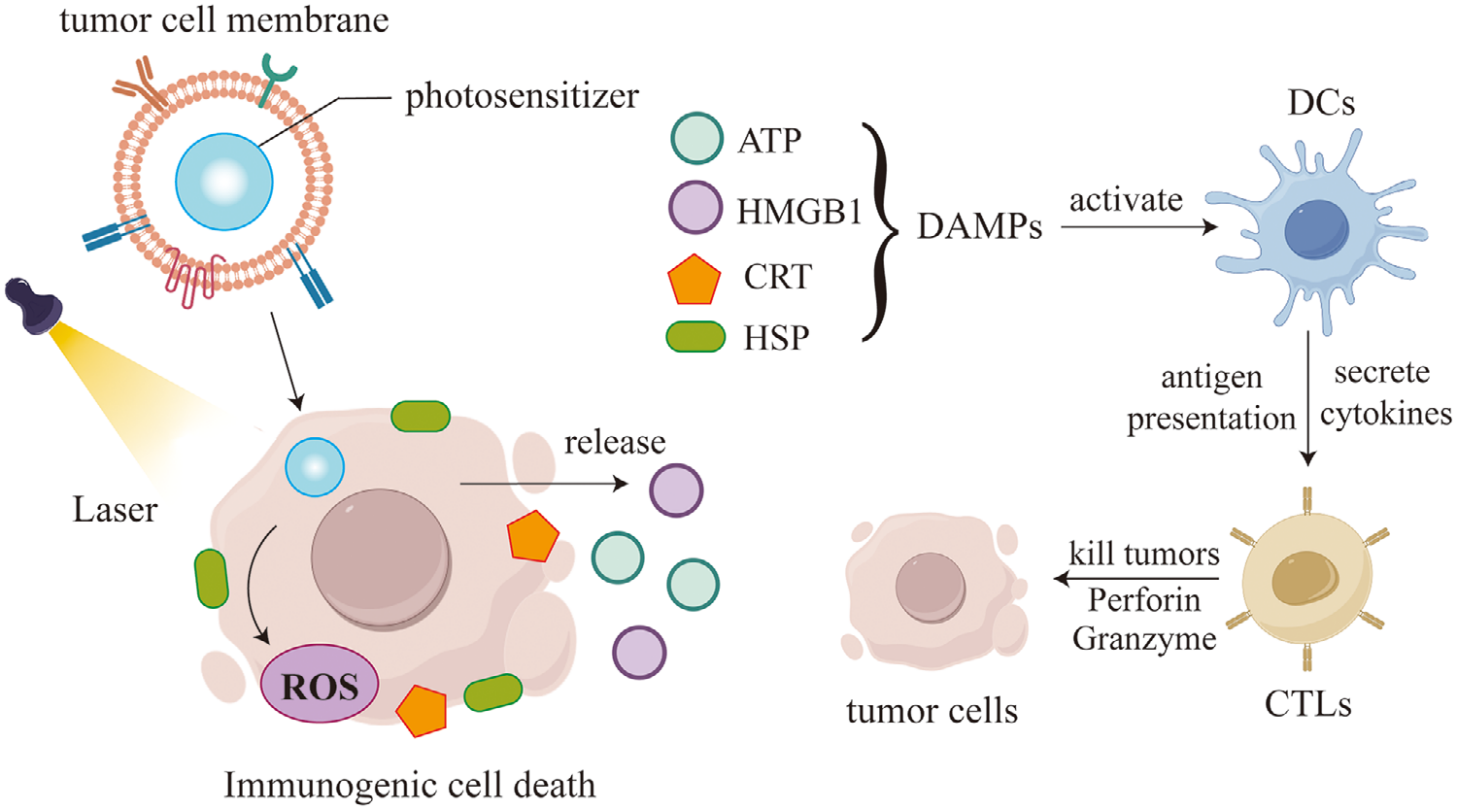
Immunogenic cell death induced in tumor cells by biomimetic nanomedicines.

Currently, ICD‐based immunotherapy has achieved great breakthroughs in clinical research, with more and more studies combining chemotherapy, radiotherapy, and PDT to synergistically enhance the therapeutic effect, while enhancing the antitumor response generated by the ICD to reduce the toxicity and side effects of the drugs (Figure 12). However, the induction efficiency of ICDs is affected by tumor type and microenvironment, and future studies need to further optimize the induction strategy and explore its clinical potential [204]. The application of biomimetic nanomedicines, with their unique biocompatibility and targeting properties, also further improves the efficacy of antitumor combination therapies, overcomes the limitations of monotherapy, and enhances the immunogenicity of tumor cells [205].

### 5.2 Nanovaccine Delivery Systems

As a groundbreaking milestone in the field of cancer immunotherapy, cancer vaccines have garnered significant attention from both scientific and clinical communities. Their mechanism involves utilizing TAAs or TSAs to elicit a tumor‐specific adaptive immune response, thereby enabling the immune system to selectively eradicate tumor cells while protecting normal tissue [206]. Based on their mechanisms of action, cancer vaccines can be classified into preventive and therapeutic vaccines. The former includes vaccines against oncogenic viruses such as human papillomavirus (HPV) and hepatitis B virus (HBV), which prevent viral infection by inducing antibody‐mediated immune responses [207]. Therapeutic cancer vaccines are designed to eliminate existing tumors, eradicate minimal residual disease, and mitigate adverse effects. Sipuleucel‐T, the first FDA‐approved therapeutic cancer vaccine for prostate cancer, operates by extracting, culturing, and engineering the own immune cells of patients, which are then reinfused to stimulate a tumor‐specific response against prostate cancer cells [208].

Cancer vaccines have demonstrated significant efficacy in oncology, while advancements in biomimetic nanotechnology have facilitated the development of novel prophylactic and therapeutic approaches. Researchers fabricated TLR7 agonist imiquimod into NPs enveloped with B16‐OVA tumor cell membranes, where membrane surface proteins serve as TSAs. Subsequent mannose modification of these NPs enhanced their uptake by APCs, thereby potentiating antitumor activation. This anticancer vaccine has been combined with ICB therapy, effectively delaying tumor progression and exhibiting promising clinical translation potential [209]. VLPs formed via the self‐assembly of Cowpea mosaic virus (CPMV) exhibit robust immunogenicity and induce macrophages to secrete abundant pro‐inflammatory cytokines. Upon inhalation by B16F10 lung melanoma‐bearing mice, these VLPs activate neutrophils to produce cytokines and chemokines, thereby triggering systemic antitumor immunity and tumor cell eradication [210].

In addition, adjuvants in cancer vaccines are a class of critical components. They typically lack antigenicity themselves but can non‐specifically enhance the intensity of the immune response against the vaccine antigen or alter the type of response [211]. Due to the weak immunogenicity of tumor antigens, which can easily lead to immune tolerance, the core function of adjuvants is to help immune cells overcome this immunosuppressive state and elicit a potent antitumor response in the body [212]. Major types of adjuvants include alum, cytokines, and nano‐carriers, among others, which can enhance the efficacy of cancer vaccines through multiple mechanisms. For instance, lipid nanoparticle carriers not only serve as delivery vehicles for mRNA vaccines, promoting the uptake of antigens by APCs, but also act as powerful immunoadjuvants themselves by activating immune pathways and generating antitumor responses [213].

**FIGURE 13:**
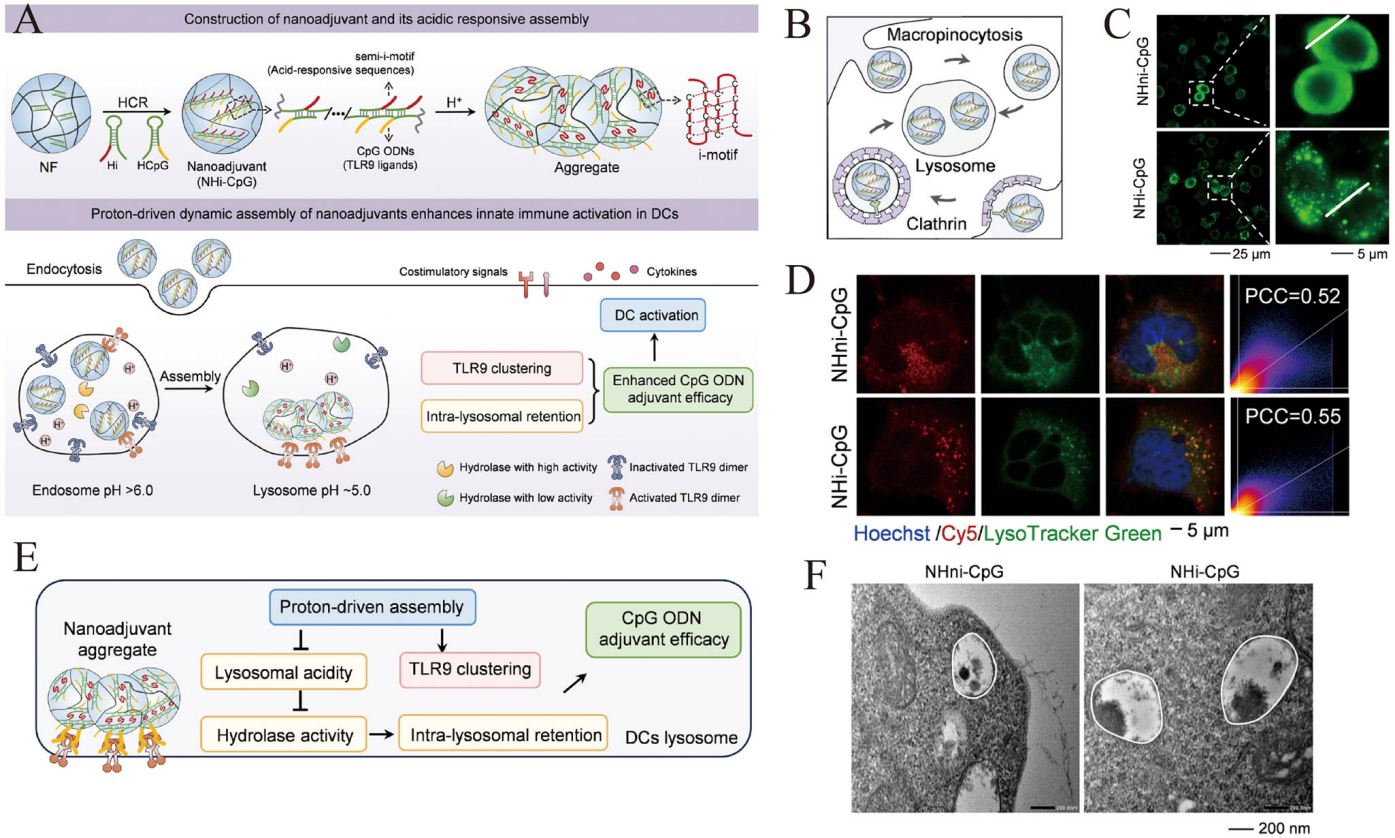
(A) A schematic diagram of DNA‐based nanoadjuvants promoting TLR9 aggregation and thereby enhancing innate immune activation. (B) Schematic diagram of the pathway for DCs to take up NHi‐CpG. (C) Immunofluorescence staining of TLR9 in DC2.4 cells with different treatments. (D) CLSM images of DCs treated with NHni‐CpG and NHi‐CpG for 4 h, followed by staining with LysoTracker Green (lysosomes) and Hoechst (nuclei). (E) The mechanism by which proton‐driven NHi‐CpG assembly promotes TLR9 clustering. (F) TEM images of DCs treated with NHi‐CpG and NHni‐CpG. Lysosomes are highlighted by white solid lines. Reprinted with permission from Ref. [214]. Copyright (2025), American Chemical Society.

Lv et al. developed a DNA‐based nanoadjuvant (NHi‐CpG) containing TLR9 ligands and cytosine‐rich DNA sequences. By activating the TLR9 signaling pathway, it can promote the activation of DCs and the proliferation of CD8+T cells, generating a powerful antitumor response (Figure 13A) [214]. Figure 13B suggested that Cy5‐NHi‐CpG was primarily internalized through clathrin‐mediated endocytosis and micropinocytosis. In the DCs treated with NHni‐CpG and NHi‐nCpG, the intensity of green fluorescence increased significantly, indicating that CpG specifically bound to TLR9 (Figure 13C). After incubating DCs with Cy5‐Nhi‐CpG and Cy5‐NHni‐CpG for 4 h, confocal laser scanning microscopy (CLSM) observed that both Nhi‐CpG and NHni‐CpG were co‐localized with lysosomes (Figure 13D). Figure 13E showed the mechanism by which proton‐driven NHi‐CpG assembly promotes TLR9 clustering. In the TEM images of DC, spherical nanostructures were observed in lysosomes far from the lysosomal membrane in the NHni‐CpG group, while DC treated with NHi‐CpG aggregated in lysosomes close to the lysosomal membrane (Figure 13F).

### 5.3 Enhanced Immune Checkpoint Blockade (ICB)

Immune checkpoints are a class of immunosuppressive molecules expressed on immune cells that are responsible for maintaining immune tolerance and preventing autoimmunity. CTLA‐4 and PD‐1, which are present on the surface of T cells, can bind to the corresponding ligands and produce co‐inhibitory signals to inhibit the antitumor activity of T cell activity, which promotes the evasion of tumor cells [215]. ICB therapy is one of the revolutionary breakthroughs in the field of tumor therapy in recent years, the core of which lies in targeting the immune checkpoints on the surface of immune cells, blocking inhibitory signals, reversing the immunosuppressive state of the TME, and activating the immune system to kill tumors [4]. Immune checkpoint inhibitors (ICIs), represented by PD‐1/PD‐L1 inhibitors, have brought clinical remission to patients with a wide range of solid tumors and hematologic malignancies, changing the cancer treatment landscape. A large number of clinical trials have shown that ICI can help improve the condition of patients with tumors. KEYNOTE‐671 was a randomized, double‐blind, global phase 3 trial that demonstrated that the addition of perioperative pembrolizumab (anti‐PD‐1) to neoadjuvant chemotherapy significantly improves event‐free survival in patients with early‐stage non‐small cell lung cancer [216].

**FIGURE 14:**
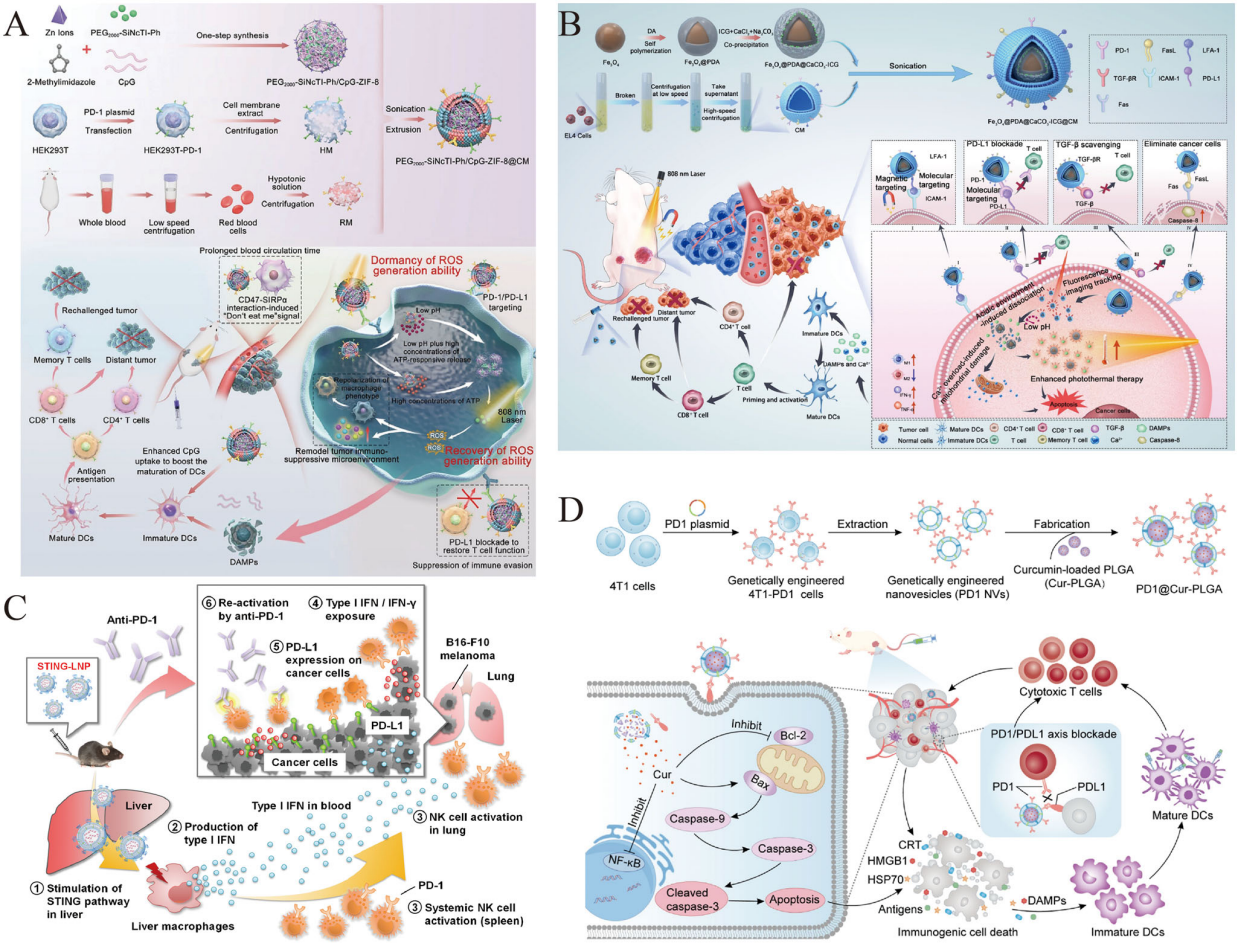
(A) Schematic diagram of PEG2000‐SiNcTI‐Ph/CpG‐ZIF‐8@CM for precision photodynamic immunotherapy of CT26 colon cancer. Reprinted with permission from Ref. [217]. Copyright (2024), Wiley. (B) Schematic of Fe3O4@PDA@CaCO3–ICG@CM for Dual Tumor Targeting‐Assisted Multimodal Therapy. Reprinted with permission from Ref. [218]. Copyright (2024), American Chemical Society. (C) Summary for reducing anti‐PD‐1 resistant by STING‐LNP. Reprinted with permission from Ref. [219]. Copyright (2021), BMJ. (D) Schematic illustration of the antitumor immune response mechanism of PD1@Cur‐PLGA. Reprinted with permission from Ref. [220]. Copyright (2024), Ivyspring International.

Recently, the combination of biomimetic nanomedicine and ICB therapy has become a brand‐new direction in tumor immunotherapy, the core of which lies in the use of nanotechnology to enhance drug targeting, reduce toxicity, and synergistically regulate the TME, which is expected to significantly enhance the antitumor efficacy. The photosensitive biomimetic nanoplatform (PEG2000‐SiNcTI‐Ph/CpG‐ZIF‐8@CM) is encapsulated in a fusion cell membrane, with its surface overexpressing PD‐1 [217]. It is activated in the acidic and high ATP environment of tumors, triggering ICD of CT26 tumor cells through PDT, which is helpful for the precise treatment of colon cancer (Figure 14A). The multifunctional biomimetic nanoplatform (Fe3O4@PDA@CaCO3‐ICG@CM) is encapsulated by the membrane of mouse lymphoma cells and can effectively target CT26 colon tumor cells [218]. Under the acidic TME, the combination of PDA and ICG enhances the photothermal effect, and can also induce tumor cell apoptosis through the direct killing effect of FasL. Furthermore, PD‐L1 blockade and TGF‐β are used to further enhance antitumor immunity (Figure 14B). Resistance to ICIs is a major obstacle to cancer immunotherapy. Lipid NPs containing interferon gene STING agonists (STING‐LNP) can be combined with anti‐PD‐1 to produce IFN‐ γ by activating NK cells, resulting in increased expression of PD‐L1 in cancer cells, reducing drug resistance and enhancing antitumor activity [219] (Figure 14C). Liao et al. developed a genetically engineered nanoparticle (PD1@Cur‐PLGA), overexpressing PD‐1 and loaded with curcumin (Cur), integrated ICD and ICB therapies to enhance tumor immunotherapy [220]. The NPs can block PD‐L1 on the tumor surface, thereby improving the cytotoxicity of T cells to tumor cells. Cur can effectively induce the release of ICD and DAMPs, promote the maturation of DCs, and the activation of cytotoxic T cells by activating caspase and Bax apoptotic signaling (Figure 14D).

**FIGURE 15:**
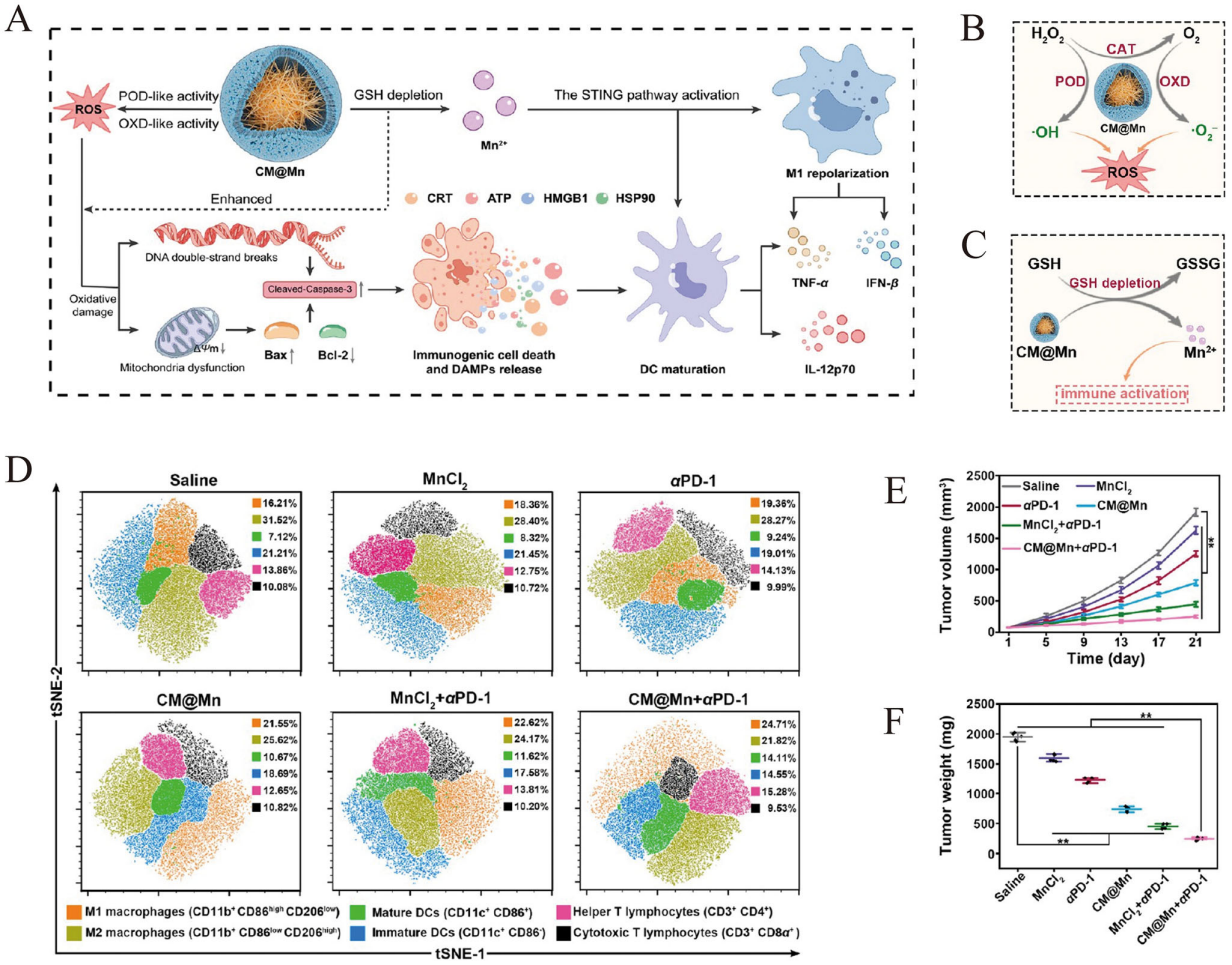
(A) Schematic illustration of ICD and immune activation mechanism mediated by CM@Mn nanozyme. (B) Schematic illustration of the catalytic mechanism of CM@Mn nanozyme. (C) Schematic illustration of GSH depletion of CM@Mn nanozyme. (D) Representative t‐SNE analysis plots of intratumoral infiltration of immune cells on day 21. (E) Average tumor growth curves of primary tumors after various treatments. (F)Weight of the dissected primary tumors after various treatments on day 21. Reprinted with permission from Ref. [221]. Copyright (2022), American Chemical Society.

As shown in Figure 15A‐C, manganese oxide nanozymes encapsulating tumor cell membranes exhibit peroxidase and oxidase‐like activity in acidic TME, producing ROS to kill tumor cells and cause ICD. In addition, Mn^2+^can promote DCs maturation and macrophage polarization into M1 phenotype, reversing the immunosuppressive TME. The membrane also contains PD‐1 monoclonal antibody (aPD‐1), further stimulating antitumor response [221]. Randomly divide the mice into six groups and administer saline or medication to monitor tumor growth and weight, and the CM@Mn + aPD‐1 group showed the greatest tumor suppression effect (Figure 15E,F). The t‐distribution stochastic neighbor embedding (t‐SNE) analysis chart shows the transformation of M2 to M1 macrophages and maturation of DCs after treatment with biomimetic nanozymes (Figure 15D), promoting immune activation.

**FIGURE 16:**
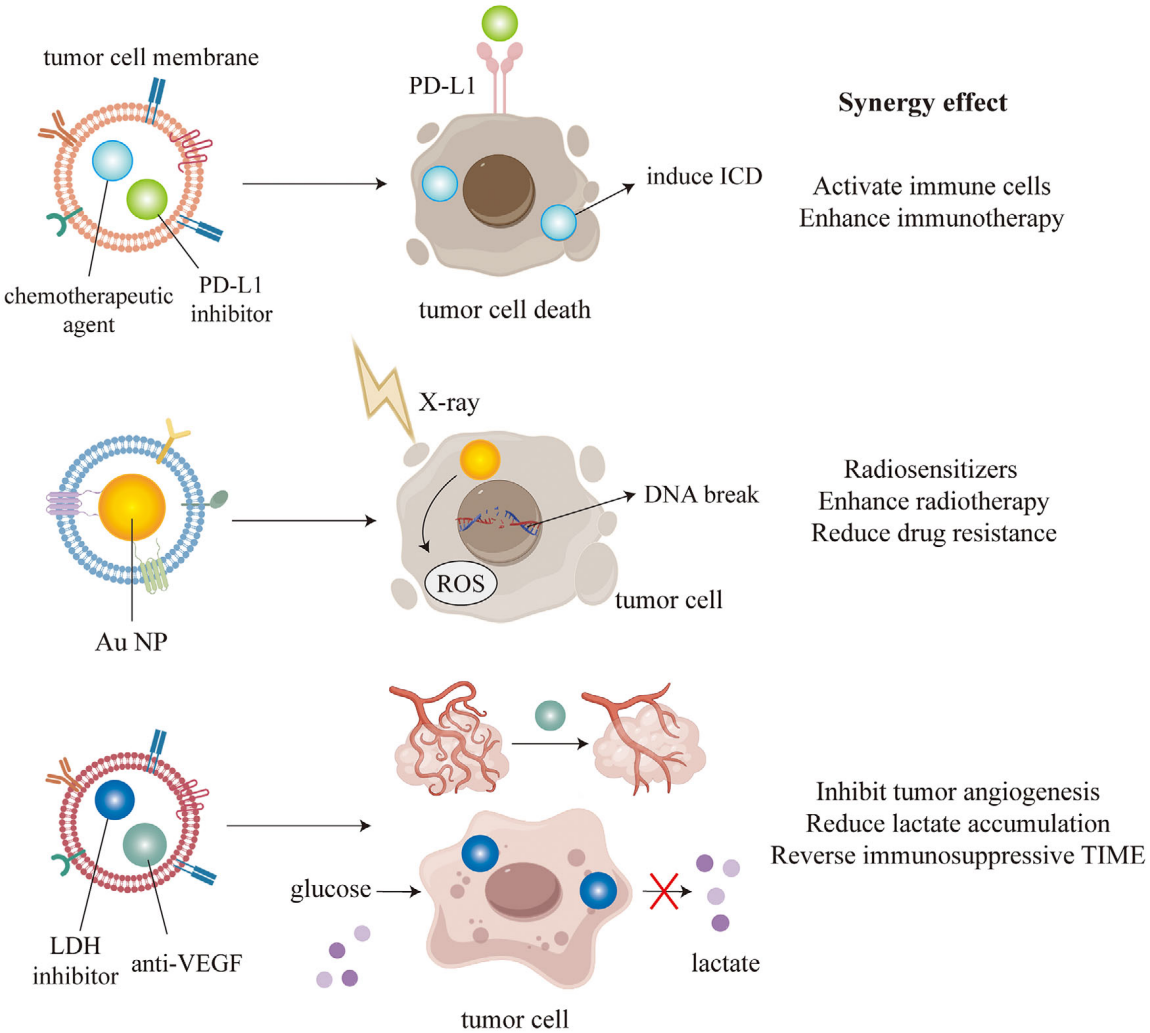
Biomimetic nanomedicines combine with antitumor therapy for synergistic effects.

It should not be ignored that ICB therapy also has many drawbacks, such as low actual patient response rate, immune‐related adverse events, and drug resistance [222]. Therefore, more advanced methods need to be explored to improve efficacy and combine with biomimetic nanomedicines to enhance antitumor effects and reduce the occurrence of side effects (Figure 16).

## 6 Prospects and Challenges of Future Research

The complexity of the TME presents significant challenges for antitumor therapy. Biomimetic nanomedicine simulates natural biological components to achieve targeted drug delivery, while regulating immune cell function and enhancing antitumor response [223]. The therapeutic superiority of biomimetic nanomedicines stems directly from their emulation of biological principles. Functionally, the therapeutic advantages of biomimetic nanomedicines in antitumor therapy lie in their capacity for specific targeting, immune evasion, and multidimensional functional integration. Erythrocyte membrane‐camouflaged nanomedicines show enhanced biocompatibility and prolonged plasma half‐life through surface‐expressed immune molecules such as CD47, evading host immune clearance [115]. Immune cell membrane‐derived biomimetic NPs not only achieve immune escape but also exhibit intrinsic tumor‐targeting capabilities, significantly improving drug enrichment efficiency within tumor tissues [96]. Crucially, these bio‐engineered platforms enable comprehensive therapeutic enhancement through precise modulation of certain components in TME, including reprogramming immunosuppressive cells and normalizing pathological microenvironmental conditions [224]. For example, using macrophage membrane‐coated nanocarriers can actively recognize and penetrate into TAMs‐enriched regions, reversing the pro‐tumor M2 phenotype to antitumor M1 phenotype through specific drugs, and enhancing antitumor effects [225]. These mechanisms synergistically enhance the infiltration and functional activation of effector T cells and NK cells, overcoming immunosuppressive barriers [37]. Table 2 summarizing the mechanisms and advantages and disadvantages of various strategies for modulating the TME, and how the disadvantages can be remedied with biomimetic nanomedicines.

**TABLE 2:** Comparison of TME regulation strategies.

Regulatory strategy	Mechanisms	Advantages	Limitations	Nanomedicine Solutions	Refs.
Immune checkpoint inhibitors (ICIs)	Blocking the inhibitory signaling pathway of immune cells, restoring the activation and proliferation of T cells, and enhancing antitumor immunity	1) a wider range of indications for a variety of tumors 2) lower toxicity compared to chemotherapy, and some patients can achieve long‐term survival	1) low overall effectiveness, some patients do not respond 2) may trigger immune‐related adverse effects and drug resistance	Biomimetic nanomedicines can co‐load ICIs with other drugs, such as chemotherapeutic agents and photosensitizers, modulating the TME by combining multiple therapies to achieve precise drug release and reversal of drug resistance	[279, 280]
Adoptive Cell Transfer Therapy	Genetically engineered T cells or NK cells to specifically recognize tumor antigens and directly kill tumor cells	1) targeting tumor‐specific antigens, reducing damage to normal tissues 2) some of the modified T cells can survive for a long TME and provide immune protection.	1) dense mesenchyme of solid tumors hinders immune cell infiltration 2) complex preparation and costly treatment 3) immunosuppressive microenvironment influences immune cell activity and function	Using biomimetic nanomedicines to improve the hypoxic and acidic tumor environment and degrade the tumor extracellular matrix (ECM) to promote immune cell infiltration	[281, 282]
Photodynamic Therapy (PDT)	Induce tumor cells to undergo immunogenic death (ICD) by generating reactive oxygen species (ROS) through photosensitizers, activating immune cells to enhance antitumor immunity	1) accurate killing of tumor cells with little damage to surrounding normal tissues 2) broad indications and minimal invasive	1) limited depth of light penetration makes it difficult to treat deep tumors 2) hypoxic environment inhibits ROS generation 3) may cause photoallergic reaction	Improving oxygen supply to the TME using biomimetic nanomedicines and delivering photosensitizers and drugs deep into the tumor for internal luminescence	[283, 284]
Sonodynamic Therapy (SDT)	Activation of acoustic sensitizers by ultrasound to generate ROS, and induces tumor cells to undergo ICD, activating immune cells to enhance antitumor immunity	1) high tissue penetration and targeting, low systemic toxicity 2) non‐invasive, low drug resistance, repeatable treatment	1) acoustic sensitizers are delivered inefficiently and do not easily accumulate in tumors 2) limited activity of acoustic sensitizers, and difficulty in developing new acoustic sensitizers	Improving delivery efficiency and tumor targeting using biomimetic nanoparticle‐loaded acoustic sensitizers. Enhancing antitumor effect through combination therapy using biomimetic NPs carrying ICIs	[285, 286]
Targeting immunosuppressive cells	Inhibits recruitment and function of Tregs and myeloid‐derived suppressor cells, modulates macrophage polarization, blocks immune checkpoint pathways, and restores T‐cell activity	1) Enhance antitumor immune response and reverse immunosuppressive microenvironment 2) Synergize with other therapies to enhance efficacy with little damage to normal tissues	1) dense ECM impedes drug and immune cell penetration 2) insufficient target specificity, prone to mistakenly injure normal cells, causing autoimmune reactions	Biomimetic NPs made from tumor cells or immune cells for homologous targeting and barrier penetration. Designing TME‐responsive biomimetic nanocarriers for co‐delivery of ICIs.	[67, 287]

### 6.1 Multidimensional Comparative Analysis of Nanoplatforms

Although this review focuses on the advanced capabilities of biomimetic strategies, it is crucial to compare their performance with other major categories of nanotherapeutic agents. Table 3 provides a systematic comparison across different platforms, evaluating them based on key metrics in tumor therapy.

**TABLE 3:** Comparative analysis of major nanomedicine platforms for tumor microenvironment targeting.

Platforms	Targeted efficiency	Biocompatibility	Immunogenicity	Metabolic toxicity	Drug loading capacity	Applicability across different TME	Production complexity	Refs.
**Biomimetic nanomedicine**	High (Active, biology‐driven)	High (Inherent “self” disguise)	Low (Especially with autologous sources)	Low (Biodegradable cores)	Moderate to High	Broad (Adaptable via membrane selection)	High (Require multiple steps)	[288, 289]
**Liposomes**	Moderate (Passive, EPR‐dependent)	High (Phospholipid composition)	Low (No immunogenic epitopes)	Low (Biodegradable into fatty acids)	High	Variable (Depends on vascular permeability)	Low (Well‐established)	[226]
**Metal‐Organic Frameworks (MOFs)**	Moderate (Requires surface engineering)	Moderate (Biodegradability varies)	Moderate (No immunogenic motifs)	Moderate (Depends on metal ion and linker)	High	Moderate (Poor adaptation to ECM‐dense TME)	High (Difficulty in batch consistency)	[228, 290]
**Covalent Organic Frameworks (COFs)**	Moderate (Requires surface engineering)	Promising (Metal‐free, organic)	Moderate (No foreign antigens)	Potentially Low (Metal‐free)	High	Moderate (Limited adaptation to TME)	High (Challenging synthesis)	[227]
**Quantum dots**	Low (Primarily for imaging)	Low (Heavy metal components)	Moderate (No immunogenic proteins)	High (Heavy metal leakage risk)	Low	Limited (Primarily for diagnostic applications)	Moderate (Surface modification adds complexity)	[229, 291]

Traditional platforms like liposomes offer advantages of clinical maturity and exceptionally high drug loading capacities. However, their reliance on passive enhancement of permeability and retention effects results in variable and often suboptimal targeting efficiency [226]. In contrast, biomimetic nanomedicines possess active, biologically driven targeting mechanisms, but this enhanced functionality comes at the cost of significantly greater manufacturing complexity. Metal‐organic frameworks (MOFs) and covalent organic frameworks (COFs) stand out for their exceptionally high drug loading capacities, with COFs showing great promise due to their metal‐free composition and potentially superior biocompatibility [227, 228]. Meanwhile, quantum dots offer significant advantages for imaging applications, but their documented cytotoxicity poses major obstacles for therapeutic use. By comparing different platforms, rational grounds are provided for selecting precision therapies [229].

### 6.2 Contemporary and Emerging Trends of Biomimetic Nanomedicine

#### 6.2.1 Hybrid Biomimetic Engineering

**FIGURE 17:**
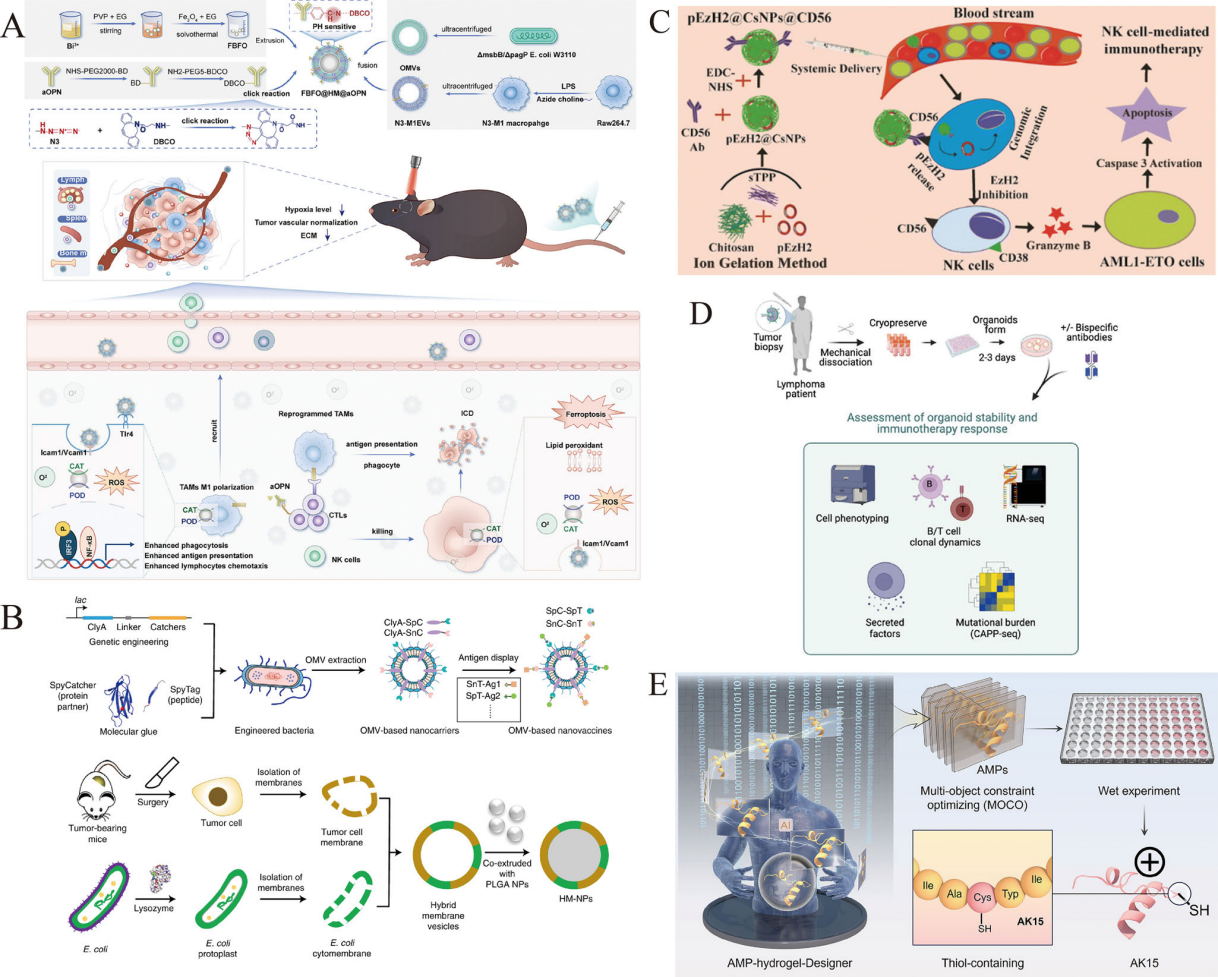
Emerging research directions in biomimetic nanomedicine. (A) Schematic illustration of the synthesis process of FBFO@HM@aOPN NPs and the treatment mechanism for immune‐remodelled PTD against glioblastoma in vivo. Reprinted with permission from Ref. [234]. Copyright (2025), Springer Nature. (B) Preparation of OMV‐ and hybrid membrane‐NPs for cancer vaccines. Reprinted with permission from Ref. [239]. Copyright (2022), Springer Nature. (C) Schematic representation of pEzH2@CsNPs@CD56 synthesis and its anti‐AML efficacy. Reprinted with permission from Ref. [243]. Copyright (2024), Royal Society of Chemistry. (D) Schematic diagram of follicular lymphoma organoid culture technology. This system enables patient‐specific modeling, high‐throughput screening, identification of TME characteristics, and assessment of therapeutic response. Reprinted with permission from Ref. [246]. Copyright (2024), Cell Press. (E) Schematic of AI‐guided AI‐AMP hydrogel design and performance. AK15 was generated and screened using the AMP hydrogel designer. Reprinted with permission from Ref. [250]. Copyright (2024), Wiley.

Single biomimetic strategies sometimes struggle to overcome all barriers within the TME, whereas hybrid biomimetic engineering achieves functional complementarity by integrating membrane components from different biological sources or synthetic materials [230]. Its core advantage lies in the ability to customize the surface properties of nanocarriers to address complex in vivo environments. The fusion forms of hybrid membranes are not limited to cell membranes (such as cancer cell membranes and immune cell membranes, RBC/platelet membranes [231]), but can also fuse cell membranes with liposome membranes [232], cell membranes with bacterial outer membranes [233], endowing biomimetic nanomedicines with multiple functions such as efficient targeting, immune escape ability, and multi‐dimensional TME reprogramming. Figure 17A depicts a multifunctional biomimetic nanoplatform (FBFO@HM@aOPN) encapsulated within a hybrid membrane formed by M1‐like macrophage‐derived exosomes and bacterial outer membrane vesicles. This platform can traverse the BBB, induce ICD in tumor cells, and reprogram TAMs toward an anti‐tumor phenotype, thereby significantly inhibiting glioblastoma growth [234]. These hybrid systems have demonstrated synergistic therapeutic effects across multiple cancer models, offering novel approaches to overcoming complex TME barriers.

#### 6.2.2 Synthetic Biology‐Enabled Nanoplatforms

Synthetic biology‐enabled nanoplatforms represent a paradigm shift in the field of biomimetic nanomedicine. At their core lies the application of genetic engineering and synthetic biology principles to redesign biological systems into programmable therapeutic tools [235]. Unlike conventional nanomedicines, these platforms transcend the limitations of static mimicry of natural structures. Through rational design and engineering, they enable dynamic and precise control over drug release, targeting localization, and immune modulation processes [236]. This capability empowers researchers to create biological components with sophisticated functions tailored to specific requirements, thereby facilitating the development of intelligent nanomedicines capable of sensing disease signals, processing biological information, and executing corresponding therapeutic functions [237].

Through rational design of synthetic pathways for DNA, RNA, proteins, and lipids, researchers can construct complex nanostructures with precise architecture and predictable functions. Concurrently, the genetic modification of mammalian cells or microorganisms enables them to secrete exosomes or membrane vesicles carrying specific therapeutic molecules, thereby establishing a viable pathway for the large‐scale production of biomimetic nanomedicines with uniform functional characteristics [238]. For instance, Researchers have developed genetically engineered OMVs and hybrid membrane vesicles containing bacterial cytoplasmic membranes for the customization of personalized cancer vaccines. Both carriers are rich in pathogen‐associated molecular patterns, enabling the activation of APCs to induce efficient antigen presentation and activate anti‐tumor immunity (Figure 17B) [239]. The significant advantage of such platforms lies in their high designability and controllability, offering immense potential for personalized therapy. However, challenges remain in large‐scale production and in vivo stability, which future technological advancements will gradually address.

#### 6.2.3 Genotype/Immunophenotype‐Specific Designs

Tumors exhibit high heterogeneity, therefore, designing targeted “Trojan horse” strategies based on a tumor's unique genetic mutation profile (genotype) and the immune cell composition of its TME (immunophenotype) aligns with the principles of precision medicine [240]. For lung cancers with characteristic EGFR mutations and TAM‐enriched pancreatic cancers, biomimetic NPs targeting these specific biomarkers can be engineered to maximize tumor cell killing while protecting normal tissues [241, 242]. Through CD56‐targeted delivery of pSMP‐EzH2 shRNA plasmid encapsulated in chitosan NPs (pEzH2@CSNPs@CD56), in vivo genetic modification of CD56‐positive NK cells was achieved (Figure 17C). This reduced EzH2 expression and activated their cytotoxic activity against leukemia cells [243]. Utilizing the patient's own tumor cells or immune cells to prepare personalized membrane coatings; or employing synthetic biology approaches to express ligands targeting specific tumor markers on the surface of nanocarriers can significantly enhance therapeutic efficacy while reducing side effects, demonstrating strong potential for clinical application.

#### 6.2.4 Spatial Immunoengineering and Organoid‐Based TME Modeling

Spatial immunology engineering and patient‐derived organoid (PDO) models provide a revolutionary platform for the preclinical evaluation of biomimetic nanomedicines. Spatial multi‐omics technologies enable the resolution of cellular spatial distribution and interaction networks within the TME at single‐cell resolution, offering unprecedented insights into the behavior of nanomedicines within complex microenvironments [244]. Simultaneously, PDO models successfully retain patient‐specific TME structures, including cellular heterogeneity, ECM composition, and physiological barriers, making them ideal tools for evaluating nanoparticle permeability and efficacy [245]. Researchers established a patient‐derived lymphoma organoid model using tumor biopsy samples from primary follicular lymphoma (Figure 17D). This model maintains stable in vitro microenvironmental conditions without exogenous cytokines for up to three weeks and reproduces T cell‐mediated lymphoma killing, enabling investigation of patient‐specific microenvironmental determinants of therapeutic response [246]. These advanced models are transforming traditional drug development workflows, enabling researchers to optimize nanoparticle design in environments closer to actual human conditions and significantly enhancing clinical translation success rates.

#### 6.2.5 AI/ML‐Guided Biomimetic Nanoparticle Optimization

Artificial intelligence (AI) and machine learning (ML) are reshaping the design and optimization processes for bionic nanomedicines [247]. By analyzing high‐throughput experimental data, AI algorithms can predict the in vivo fate and therapeutic efficacy of NPs. Deep learning models can predict the biodistribution and clearance pathways of NPs by analyzing their physicochemical parameters, such as size, surface charge and hydrophobicity [248]. The core advantage of these computational tools lies in their efficient data mining and design optimization capabilities, which significantly shorten drug development cycles and reduce trial‐and‐error costs. This provides the technological foundation for achieving truly personalized nanomedicine by integrating patient‐specific factors to recommend optimal nanomedicine regimens [249]. An AI‐based biomimetic design platform has been successfully developed for generating antimicrobial biomaterials [250]. By integrating generative design with multi‐objective constrained optimization techniques, the platform synthesizes novel thiol‐containing antimicrobial peptides (AMP) with high efficacy. These peptides are functionally coupled with hydrogels to enable targeted treatment of drug‐resistant bacterial infections (Figure 17E).

### 6.3 Challenges and Transformation Analysis of Biomimetic Nanomedicines

The heterogeneity of the TME manifests in multiple aspects, including immune cell infiltration, stromal density, vascular distribution, and metabolic status [251]. This complexity makes it challenging for a single, “one‐size‐fits‐all” nanomedicine design to be effective for all patients. Significant TME heterogeneity exists both between patients and within the same patient. Furthermore, different tumor patients receiving identical treatments may exhibit distinct TME phenotypes [252]. To address this challenge, it is essential to deeply analyze patient‐specific TME molecular profiles. By leveraging technologies such as single‐cell sequencing, spatial transcriptomics, and liquid biopsy, precision biomimetic strategies should be applied to the design of biomimetic nanomedicines, thus maximizing the mitigation of therapeutic disparities caused by TME heterogeneity [253].

The off‐target risks associated with membrane‐coated NPs primarily stem from the nonspecific binding of naturally retained surface receptors to healthy tissues, or the adsorption of complement proteins and antibody onto the membrane surface, leading to recognition and clearance by the mononuclear phagocyte system (MPS) [254]. Synthetic biology and membrane engineering offer powerful tools for precisely modulating their targeting specificity. Through genetic engineering of donor cells, proteins responsible for off‐target interactions can be knocked out while tumor‐targeting receptors are overexpressed, thereby enhancing targeting precision and reducing off‐target toxicity [255]. Additionally, reducing cholesterol levels in cell membranes, adding new modifying molecules, or incorporating multiple hybrid membranes can all decrease MPS clearance and non‐specific binding [256, 257].

The immunogenicity of biomimetic NPs and their in vivo clearance kinetics directly impact their clinical safety [258]. Although cell membrane coatings provide excellent biocompatibility, the allogeneic or xenogeneic cells, bacterial membranes, and viral envelopes may trigger undesirable immune responses. Regarding clearance kinetics, physicochemical properties such as nanoparticle size, surface charge, and membrane composition collectively influence retention in the liver and spleen, posing potential toxicity risks [259]. Countermeasures include utilizing patient‐derived autologous cells as membrane sources, designing biodegradable core materials to ensure safe metabolic clearance after drug delivery, and employing humanized PDO and animal models for early‐stage immunotoxicity assessment during development.

Beyond the aforementioned limitations of biomimetic nanomedicines, significant translation bottlenecks persist in scaling laboratory achievements to mass production and clinical application [260]. Challenges primarily center on achieving large‐scale, highly reproducible cell membrane extraction and nanoparticle coating processes, alongside the lack of preclinical models capable of accurately predicting in vivo efficacy [261]. Although lipid‐based nanomedicines have been used for decades in anticancer drug delivery, premature drug leakage before reaching tumor sites may reduce drug concentrations at tumor sites and increase off‐target toxicity risks [262]. Corresponding regulatory policies remain unclear, with no established evaluation standards for hybrid or engineered membrane materials.

To overcome these obstacles, manufacturing processes should transition from traditional batch methods to microfluidic production systems, integrated with online quality monitoring to ensure consistent and stable product quality [263]. In the evaluation system, moving beyond simple 2D cell lines and rodent models to actively employ PDOs and humanized mouse models for efficacy validation is essential. These models better recapitulate the complexity of the human TME and drug‐human immune system interactions [264]. Furthermore, regulatory science must evolve in parallel to establish review standards for these complex bio‐synthetic hybrid products.

## Funding

This work was supported by the Grants for Scientific Research of BSKY from Anhui Medical University (XJ201909), the Scientific Research Level Improvement Plan of Anhui Medical University (2021xkjT017), the Scientific Research Foundation of the Educational Department of Anhui Province (2024AH050674), and the Special Fund for Scientific Research of Wu Jieping Medical Foundation (320.6750.2025‐21‐22).

## Conflicts of Interest

The authors declare no conflicts of interest.

## Data Availability

No data was used for the research described in the article.
